# Presenting the direct intercultural effectiveness simulation: an implicit trait policy on intercultural competence

**DOI:** 10.3389/fpsyg.2023.1137871

**Published:** 2023-06-29

**Authors:** Stijn Schelfhout, Eva Derous

**Affiliations:** ^1^Vocational and Personnel Psychology Lab, Department of Work, Organisation and Society, Faculty of Psychology and Educational Sciences, Ghent University, Ghent, Belgium; ^2^Department of Experimental Psychology, Faculty of Psychology and Educational Sciences, Ghent University, Ghent, Belgium

**Keywords:** implicit trait policy (ITP), general domain knowledge, intercultural effectiveness, intercultural competence, intercultural traits, MacGuffin scenario

## Abstract

**Background:**

An implicit trait policy (ITP) represents the interaction between a personal disposition and general domain knowledge on how to effectively handle a specific (intercultural) situation. Such an ITP is a proven construct to create instruments that can predict future effective behavior. Moreover, such a simulation can provide valuable proxies for actual (future) behavior, as measures of (future) real life intercultural interactions are not always available.

**Methods:**

In a series of three studies (N_1_ = 224, N_2_ = 291, N_3_ = 478), the present research introduces a “Direct Intercultural Effectiveness Simulation” or DIES, an instrument that simulates intercultural effectiveness by directly tapping into an ITP on intercultural competence.

**Results:**

First and foremost, the present research demonstrates that the DIES instrument generates reliable and construct-valid measures of intercultural effectiveness. Second, the DIES instrument also shows expected converging and diverging patterns when correlated with a nomological network on intercultural effectiveness. And third, the DIES measure is further validated by integration into an ITP framework of intercultural effectiveness based on theoretical and empirical accounts from literature.

**Conclusion:**

The DIES instrument generates a reliable and valid measure of intercultural effectiveness by tapping into an ITP on intercultural competence. Theoretically, the present research integrates the instrument into literature by empirically verifying an ITP framework of intercultural effectiveness. In practice, the DIES instrument can be used as an awareness or training proxy for actual behavior to tackle important problems like ethnic prejudice and discrimination.

## 1. Introduction

How effectively do individuals cope with intercultural situations in key areas of human society like education (Hagenaars et al., 2023), health care (Egede-Nissen et al., 2019), housing (Ghekiere et al., 2022), and work (Lippens et al., 2022)? Summarizing this literature, Leung et al. (2014) have suggested a theoretical framework that explains intercultural effective behavior as a function of intercultural competence. Intercultural competence is described as a multifaceted concept (i.e., traits, attitudes, and capabilities) that also incorporates elements like experience, motivation, and learning to perform effectively and appropriately when interacting with individuals from a different cultural background (Dias et al., 2020). The framework was recently empirically validated (Schelfhout et al., 2022), indicating that personality drives the framework, while especially cultural capabilities lead to self-efficacy beliefs toward intercultural effective behavior.

Such trait-driven knowledge on effectiveness is especially useful toward research and instruments that study and use general domain knowledge (GDK). General domain knowledge represents common beliefs of behavior that is presumed effective across various situations (Naemi et al., 2016; Torres and Beier, 2016). A very specific form of GDK is called an implicit trait policy or ITP (Motowidlo et al., 2006; Shaw, 2021). An ITP represents self-beliefs about a causal relationship between personality traits and behavioral effectiveness (Oostrom et al., 2012). Such a self-belief is the result of the interaction between one’s personality traits (or trait constructs) and GDK of what is presumed effective in specific situations (Oostrom et al., 2019). As an example of such an interaction, showing empathy is commonly believed to have a higher chance of effectively resolving a situation in an interaction compared to non-empathic behavior. As empathy is in fact truly predictive of effectiveness (Pedersen and Pope, 2010), a personal self-belief or ITP that empathy is effective can predict future effective behavior. To operationalize such a construct, an ITP can be directly simulated by using a specific (scenario) setup in which participants have to distinguish between effective and ineffective responses (Martin-Raugh et al., 2016). To this date and to the best of our knowledge, a direct simulation of an intercultural ITP is not yet described in literature. Such a direct simulation could explain why intercultural competence can lead to intercultural effective behavior as more competent individuals should also have stronger self-efficacy beliefs about intercultural competence under the form of a more pronounced ITP and thus exhibit more effective intercultural behavior.

In a series of three empirical studies (*N*_1_ = 224, *N*_2_ = 291, *N*_3_ = 478), the present research therefore introduces a new instrument called the “Direct Intercultural Effectiveness Simulation” (or DIES). The DIES instrument consists of four scenarios taken from key areas of human society in which participants have to evaluate hypothetical responses to tense intercultural interactions toward their effectiveness. As such, the performance on the instrument simulates intercultural effectiveness by tapping into an ITP on intercultural competence. Individuals that have a higher intercultural competence (comprising of traits, attitudes, and capabilities) are thus assumed to display more interculturally effective behavior due to their self-belief that a higher competence indeed leads to more effectiveness (Leung et al., 2014). To test this assumption, the results on the DIES are integrated into existing frameworks in literature regarding GDK and ITP (Lievens and Motowidlo, 2016) and intercultural competence and effectiveness (Leung et al., 2014; Schelfhout et al., 2022).

### 1.1. Implicit trait policy

An ITP is based on the accentuation effect (Tajfel, 1957). The accentuation effect is observed when differences between-groups are exaggerated or accentuated and differences within-groups are toned down. For instance, if effective and ineffective behaviors are presented, individuals are able to distinguish the effective from the ineffective behaviors, while the internal differences of effective and ineffective behaviors, respectively, will fade (Motowidlo et al., 2006). The accentuation effect has been replicated numerous times in different areas of social and behavioral science, with effects regarding ethnicity (Corneille et al., 2004), but also economics (Dubois et al., 2010), and categorization (Sherman et al., 2009) and has thus received ample support in literature.

A direct simulation of an ITP however, considers the accentuation effect in interaction with a trait or trait construct like agreeableness. An ITP thus consists of a focal, effectiveness part and a peripheral, trait-origin part. Martin-Raugh et al. (2016) and Motowidlo et al. (2016, 2018) presented several studies in which a direct simulation of an ITP on prosocial knowledge was reported. Results showed that individuals were able to distinguish (prosocial) effective responses from (antisocial) ineffective responses. Results also showed that a person high on prosocial knowledge displayed a higher simulated (ITP) effectiveness compared to a person low on prosocial knowledge as the former (vs. the latter) will score the effective responses as even more effective, and the non-effective responses as even more non-effective. In other words, a high ITP for prosocial knowledge provides a link between prosocial knowledge and effective behavior.

For the present research on the DIES instrument, we therefore consider an ITP on intercultural competence, in which intercultural competence (i.e., traits, attitudes and capabilities) interacts with an accentuation effect on intercultural effectiveness as theorized by Leung et al. (2014). As we already know that intercultural competence indeed predicts intercultural effectiveness, a personal self-belief or ITP that intercultural competence is effective can predict future effective behavior (Schelfhout et al., 2022). Such a specific ITP setup has consequences for validation.

Traditionally, an ITP is validated by observing the correlation between the trait dimension (e.g., cultural empathy) and the effectiveness of the ITP of said trait dimension (e.g., cultural empathy ITP) (Motowidlo et al., 2006). This correlation is called the saturation of the ITP, which is not always a very strong one (Freudenstein et al., 2023). As a consequence, some accounts in literature have criticized this traditional approach, clearly stating that the variance of an ITP over individuals is not determined by personality alone, but can also include elements like experience, attitudes, motivation and learning (Oostrom et al., 2012; Lievens and Motowidlo, 2016). Moreover, the single trait—single ITP setup is also criticized, as personality dimensions are known to correlate, which complicates the construal of personality instruments (Corstjens et al., 2017). Instruments that leave out one or more known dimensions of more broad theoretical concepts (e.g., the Big Five of human personality) can endanger the validity of correlation and regression estimates through the omitted variable problem, as the estimated relations are prone to bias (i.e., in the present research’s case an underestimation of saturation) by leaving important elements (e.g., one or more personality dimensions or elements of experience, attitudes, motivation, and learning) out of the equation (Sackett et al., 2003). As a consequence, there are already accounts in literature that disavow the (single) trait specificity of the ITP construct (Freudenstein et al., 2023).

**Figure 1:**
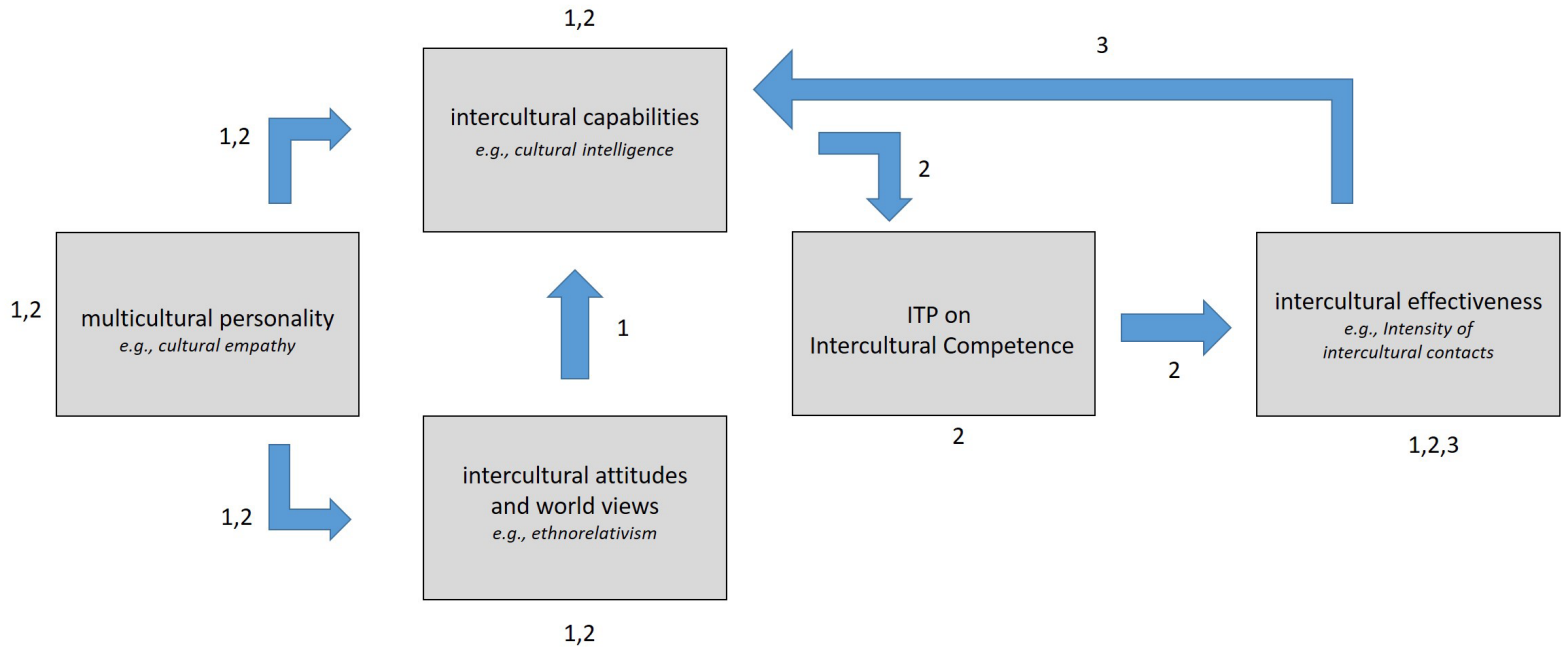
The implicit trait policy framework of intercultural effectiveness. This figure integrates the theoretical accounts of Leung et al. (2014) (components and relations annotated with a 1) and Shaw (2021) (components and relations annotated with a 2) into the ITP framework of intercultural effectiveness, with an added feedback component as suggested by SCCT (Lent et al., 1994), components and relations annotated with a 3. A high disposition on intercultural traits (e.g., cultural empathy, see van Oudenhoven and van der Zee, 2002) predicts both an ethnorelative attitude (Bizumic and Duckitt, 2012) as well as more intercultural capabilities (e.g., cultural intelligence, see Schwarzenthal et al., 2019). An ethnorelative attitude also predicts more intercultural capabilities. The ITP on intercultural competence is positioned between intercultural capabilities and intercultural effectiveness (e.g., intensity of intercultural contacts, see also Schwarzenthal et al., 2020). Finally, intercultural effectiveness also feeds back into intercultural capabilities.

A full Big Five trait—driven framework, also incorporating aforementioned elements like experience, attitudes, motivation, and learning would therefore be a more robust construct to explain the origin of an ITP in comparison to a single personality dimension and determine its saturation. Such a setup makes sense, as (the absence of) behavior as a reaction to a situation can emerge as the result of the interaction between possible antecedents like the full spectrum of human personality and elements like experience, attitudes, motivation, and learning (Ackerman, 1996). For the present research, we thus aim to validate the DIES instrument as a direct simulation of intercultural effectiveness by tapping into an intercultural competence ITP. The origin of the ITP regarding traits, attitudes and capabilities is explained through the ITP framework of Intercultural Effectiveness (see also Figure 1).

### 1.2. The implicit trait policy framework of intercultural effectiveness

The present research integrates two theoretical accounts in literature to validate the results of our DIES instrument as a measure of intercultural effectiveness by tapping into an ITP on intercultural competence. The first account is the framework of intercultural competence by Leung et al. (2014). The second account is the model by Shaw (2021) on how GDK as an ITP can lead to more effective behavior. As both accounts already summarize a vast amount of literature on intercultural competence and ITPs respectively, our intention is not to question these accounts and replicate the results of all hypotheses made. Instead, we merely want to integrate these accounts into an ITP framework of intercultural effectiveness. This framework can then be used in our analyses to add to the validation of our instrument as a measure of intercultural effectiveness. For further reading on both accounts, we refer to Leung et al. (2014) and Shaw (2021).

Considering the first account, Leung et al. (2014) suggested a theoretical framework that explains intercultural effectiveness as a function of intercultural competence. Intercultural effectiveness represents how effective an individual behaves when interacting with other, culturally diverse individuals (Nesdale et al., 2012). Intercultural effective behavior can result in beneficial outcomes like improved contacts and cooperation (Schwarzenthal et al., 2020). Intercultural competence has three components that interact in a specific way toward intercultural effectiveness: intercultural personality traits like cultural empathy (i.e., how one feels about others in intercultural situations) (van Oudenhoven and van der Zee, 2002; van der Zee et al., 2013), intercultural world views and attitudes like ethnocentrism (i.e., an attitude that considers only one, superior culture) or ethnorelativism (i.e., an attitude that considers many cultures of equal worth) (Ng et al., 2009; Bizumic and Duckitt, 2012; Young et al., 2017) and intercultural capabilities (i.e., which personal strengths does one have to navigate intercultural situations?) like cultural intelligence (Schwarzenthal et al., 2020). The framework also posits four relations between the different components, that have received ample support in literature: (1) high trait dispositions predict an ethnorelative attitude, (2) high trait dispositions predict more intercultural capabilities, (3) an ethnorelative attitude predicts more intercultural capabilities, and (4) more intercultural capabilities predict higher intercultural intercultural effectiveness.

Considering the second account, Shaw (2021) theorizes that GDK on effectiveness like an ITP can originate as a form of effective actions toward problem solving, through a number of key processes. Applying the ITP origination to the context of the present research, intercultural competence can thus generate an ITP through three subprocesses. As a first process, (1) GDK is context-independent, commonly known information about which responses are effective in a specific domain (Krumm et al., 2015). For instance, literature already indicates that consensus-based measuring (CBM) ratings of layman can match subject matter expert (SME) ratings for intercultural competence (Osmancevic et al., 2021). A recent account in literature also showed that individuals report a high self-efficacy regarding the intent to use intercultural effective behavior in intercultural situations (Schelfhout et al., 2022). Self-efficacy is arguably considered the most important predictor of actual behavior according to Social Cognitive Career Theory or SCCT (Lent et al., 1994; Stajkovic and Luthans, 1998). As a second process, (2) GDK (partly) originates from personality that drives the process of intellect development (Ackerman, 1996). For the present research, literature indeed shows that multicultural personality traits drive intercultural world views and attitudes (Nesdale et al., 2012; Horverak et al., 2013), and intercultural capabilities (Ang et al., 2006). As a third process, (3) accumulated knowledge (i.e., an ITP as GDK) can function both as an antecedent as well as a consequence. In such a way, knowledge from how to handle one (prior) situation effectively, can help in handling future situations effectively. If we consider integrating the processes in the framework by Leung and colleagues, the ITP on intercultural competence should be placed in between capabilities and effectiveness, as this pathway is the only theorized connection between competence and effectiveness (see above).

To this day and to the best of our knowledge, it is remarkable that both ITP—literature as well as intercultural effectiveness literature do not yet model feedback of actual behavior back into the origins of that behavior. Indeed, elements like knowledge should work as both an antecedent as well as a consequence to behavior (Ackerman, 1996). However, there are accounts in literature like SCCT that do feature models in which elements like performance have an effect that feeds back into the origins of that behavior (Lent et al., 1994; Stajkovic and Luthans, 1998). As an ITP draws on elements like the knowledge available, we therefore add a variable to the present research that is able to simulate the feedback effect of effectiveness on capabilities. We therefore opt to include the intensity of intercultural contacts (IOC) in the present research as a proxy for intercultural effectiveness. Although the number or the intensity of contacts is already linked to intercultural effectiveness in several accounts in literature (Schwarzenthal et al., 2019, 2020; Piekut, 2021), other accounts also consider IOC an antecedent to acquiring further intercultural capabilities (Sousa et al., 2019; Lin and Shen, 2020).

In sum, Figure 1 integrates (1) the accounts on an ITP as GDK (Lievens and Motowidlo, 2016), (2) intercultural effectiveness (Leung et al., 2014; Schelfhout et al., 2022), and (3) the possibility of feedback from SCCT (Lent et al., 1994) into a framework that explains how the DIES as a direct simulation of intercultural effectiveness taps into an ITP on intercultural competence. Over the course of three studies, the present research will fit this ITP framework of intercultural effectiveness to the acquired data.

Finally, we acknowledge the existence of concepts and subsequent scales that are also linked to or can predict intercultural effectiveness like modern racism (McConahay et al., 1981). For sure, these scales also show merit toward predicting intercultural competence and effectiveness, although usually not supported by a full theoretical framework. Still, we opt to act conservatively and also incorporate a battery of these scales as a nomological network that can establish congruent validity for the DIES as a measure of intercultural effectiveness. For a full description of the measures used, we refer to the Methods and materials sections below.

### 1.3. Present research

The present research is conducted within a project funded by the national research foundation to chart and remedy discrimination in four constitutionally anchored areas of human society: education, healthcare, housing, and work (Ghekiere et al., 2022; Hagenaars et al., 2023; Lippens et al., 2022; Schelfhout et al., 2022). The project focuses on improving the intercultural effectiveness of individuals and practitioners without migration background, toward clients in these areas who do have a migration background. The project received proper ethical approval from the responsible ethics committee (S004119N). All participants agreed to an informed consent (available as Supplemental material S1) prior to partaking in the present research.

The present research features a series of three studies to introduce and validate the DIES instrument that measures intercultural effectiveness by tapping into an ITP of intercultural competence. Throughout these studies, the fit between our ITP framework of intercultural effectivity and the empirical data acquired is assessed gradually. In Study 1, the construction of the DIES instrument is explained and assessed regarding reliability and construct validity. In Study 2, the congruent and divergent validity of the DIES scores is assessed using a nomological network on intercultural effectiveness. This network also contains an operationalization of intercultural competence, with not only traits, but also attitudes and capabilities, as it is advocated in literature to explore the origin of an ITP (Lievens and Motowidlo, 2016). We operationalized capabilities using cultural intelligence (i.e., CQ), which is not merely a measure of pure knowledge, learning or experience, but more specifically comprises of dimensions of (meta-) cognition, motivation and behavior (Schwarzenthal et al., 2019). Finally, Study 3 investigates to which extent individuals intend to use interculturally effective behavior toward intercultural situations. The DIES results are validated using a structural equation modeling (SEM) on the ITP framework of intercultural effectiveness. The extent to which a higher intercultural competence leads to a more pronounced ITP (i.e., saturation) is estimated using a multiple correlation coefficient, which is operationalized as the square root of the explained variance R^2^ from a linear regression.

## 2. Study 1: direct simulation of intercultural effectiveness knowledge

The goal of the DIES instrument is to simulate how effective individuals respond to intercultural situations by accentuating the differences between intercultural effective and ineffective responses through tapping into an ITP on intercultural competence. To ensure the best possible outcome regarding reliability and validity, the practical implementation of the construct-driven instrument follows the review by Corstjens et al. (2017) on situational judgment tests (SJT) regarding (1) item stems, (2) item response options, instructions and format, and (3) scoring key. However, as the present instrument also adheres to the theoretical setup suggested by Ackerman (1996), Shaw (2021), and Leung et al. (2014), we do not consider the DIES what is called a construct-driven SJT focusing on a single trait origin, but rather as a direct simulation of intercultural effectiveness, tapping into an ITP on intercultural competence.

The materials for the item stems and response options were obtained using a procedure of co-creation within four fields of interest including education, healthcare, housing, and work. Although not strictly needed for the construction of theory-driven SJTs (Corstjens et al., 2017), critical incidents were gathered from interest groups regarding the rights of ethnic minority patients within the four fields of interest to function as the base for our scenario stems and item responses (for more information on critical incidents, see also Herfst et al., 2008). The draft of the scenarios and the responses based on these incidents was then presented to a panel of SME in their respective field to control for realism and face validity. The final version was sent back to the interest groups for final approval.

For the item stems, intercultural competence should feature five trait dimensions that drive the model toward intercultural world views and intercultural capabilities. Indeed, according to Shaw (2021) the origins of an ITP are driven by a full spectrum of human personality. On the one hand, we consider it unwise to implement all components in one scenario as it becomes impossible to determine which component exactly activates the intercultural competence construct. On the other hand, implementing only one trait is quite limiting and even concerning, as intercultural competence can possibly be driven by all five traits, as was described in the introduction (van der Zee and van Oudenhoven, 2000; Sackett et al., 2003; Corstjens et al., 2017). As such, we opted to move the driving components from the scenario to the responses. In the past, similar instruments have been constructed without scenarios as such, as the scenario was found to be redundant in some cases (Krumm et al., 2015; Schäpers et al., 2020b). However, in some research the construal of the scenarios did affect the outcome (Freudenstein et al., 2020). For the present research, we thus opted to construct the scenario according to the MacGuffin principle from popular cinema (see also www.britannica.com/art/MacGuffin). Often attributed to Alfred Hitchcock, a MacGuffin is an element in a story or a movie that drives or initiates the action but of which the further function is limited or non-existent. Other than setting the stage for a tense intercultural interaction, we thus constructed four scenarios that harbor as little information as possible and were kept gender neutral. In such a way, a participant has to resort to its own strengths like traits, experience, attitudes, motivation, and learning regarding intercultural actions, which are summarized by the participant’s intercultural competence (see also introduction). More competent participants adhere to more intercultural effective behavior and thus have a higher chance of basing their answers on their competence, which is in line with ITP—research (Motowidlo et al., 2006).

The setting always puts an individual in a tense intercultural situation. These situations are based on (minority) reports of critical incidents by interest groups, on the topics of education, health care, housing, and work, respectively. These topics are considered basic human rights and are constitutionally anchored areas of human society in Flanders, Belgium. As an example of such a scenario^1^ (translated from Dutch, work scenario):

*“L. L. heads a well-known interim office in Flanders, Belgium. L.L. has seen a change in the multicultural character of the temp pool in recent years. Just over half of the temporary workers now have a non-native background (skin color, nationality, and religion), while most recruiters have a typically native background. The results of the selection procedures show that some recruiters systematically prefer temporary workers with a typically native background. Temporary workers with a typically native background also remain employed longer by the companies where they are outsourced than temporary workers of non-native origin. At a meeting with all recruiters, L. L. is asked how the agency will tackle this problem.”*

For the item response options, we based the responses for each scenario on five multicultural personality traits that trigger intercultural competence in line with the theoretical suggestions of Shaw (2021) as traits are theoretically and empirically considered the triggering elements of intercultural competence (Schelfhout et al., 2022). As each scenario must balance effective and non-effective responses to accentuate effectiveness, each personality trait triggers an effective and a non-effective response. These responses were again based on (minority) reports of critical incidents by interest groups, on the topics of education, health care, housing, and work. After feedback from SME (i.e., doctoral students and researchers) on further minority reports, each scenario thus features 10 revised responses. We created optimal conditions to evoke the accentuation effect as all 10 responses were presented on screen simultaneously, in randomized order. A full DIES battery thus features four scenarios with 10 responses each. However, the scenarios should be viewed as (response) items in their own right as all personality dimensions are represented in each scenario, as was addressed in the Introduction. Moreover, although elements like attitudes, experience, motivation, and learning are not explicitly targeted, the participant will make use of said elements to judge the effectiveness of the (trait-based) item responses. Of course, this use is merely an assumption and must be tested as such, especially through the saturation (i.e., the multicorrelation between the elements of intercultural competence and the DIES ITP). As an example of an effective response (translated, work scenario), “*We must first try to understand why temporary agency workers with a non-native background are employed for a shorter period of time than agency workers with a native background*.” (based on cultural empathy) and an ineffective response (translated, work scenario), “*People’s cultural background is irrelevant. The best power should always get the job*.” (based on cultural empathy). The length of effective and ineffective responses was balanced across a scenario.

For the item instructions, we considered two questions.^2^ For Study 1 and 2, we were interested in the effectiveness of responses which resulted in the question: “*Judge the effectiveness of the following responses*” (translated from Dutch). For Study 3 we were interested in participants’ intentions, which resulted in the question: “*How large is the chance you would act according to the following responses?*” (translated from Dutch).

For item response format, we presented the participants a score from 0 to 100 (with anchors very ineffective to very effective for Studies 1 and 2 and with anchors very unlikely to very likely for Study 3). The continuous response format was used to counter min-max (i.e., extreme) responding, to create more variance in the answers and to give participants a sense of agency, ultimately aiming at obtaining a more consistent instrument (Voutilainen et al., 2016).

For the scoring keys, DIES is scored as an accentuation effect, analogous to Martin-Raugh et al. (2016). The focus of scoring is on the scenario level as item responses within one scenario merge into one ITP response to the scenario, a response based on the participants’ own intercultural competence. For an ITP scenario score, the sum of the scores on the ineffective responses is deducted from the sum of the scores on the effective responses and divided by five. As the individual items are scored on a scale of 0–100, an ITP scenario score theoretically ranges from −100 to +100. The ITP scenario score is to be interpreted as the mean difference between the effectiveness judgments of effective and ineffective responses. The total ITP score is the mean of the four ITP scenario scores and represents the ITP across scenarios.

### 2.1. Methods and materials

#### 2.1.1. Data

Study 1 ran online in the Fall of 2020, as the questionnaire link was spread out amongst senior sociology students who could participate as a part of the lectures on intercultural effective behavior. The obtained dataset featured the data of *N*_1_ = 224 students, at a completion rate of 81%, of which the vast majority (i.e., about 84%) was born between 1997 and 2001, with about 58% indicating a female gender orientation. About 8% of the students indicated a personal migration background, or a father or (grand) mother with a migration background. About 17% was oblivious to this information or chose not to disclose their background. The sample thus represents the (future) target population of the overarching project.

#### 2.1.2. Measures

Means, standard deviations and reliabilities for the present research are reported separately in the Results section.

##### 2.1.2.1. Intercultural effectiveness

Intercultural effectiveness is directly simulated and scored as an ITP using the DIES instrument, according to the scoring key explained above. For Study 1, participants were asked to rate the effectiveness of the responses.

#### 2.1.3. Analyses

The reliability of the ITP scores from the DIES instrument is analyzed using a Cronbach’s alpha (from minimally acceptable *α* > 0.65 to optimal *α* > 0.80, see also Nunnally, 1978). The construct validity of the DS-DIES is evaluated with a confirmatory factor analyses (CFA) by means of SEM. The SEM model is evaluated using an indices battery including a (very conservative) chi-squared test (*χ*^2^, with *p* < 0.05 for an outstanding fit), the Comparative Fit Index (CFI, >0.95 for a good fit, >0.90 for an adequate fit), the Root Mean Square Error of Approximation (RMSEA, <0.01 for an excellent fit, <0.05 for a good fit, <0.08 for an adequate fit) and the Standardized Root Mean Square Residual (SRMR, <0.08 for a good fit) (Schreiber et al., 2006; Ullman and Bentler, 2012). Note that the cutoffs are rules of thumb; an evaluation of a SEM model should primarily observe the general pattern rendered by all indices (see also http://www.davidakenny.net/cm/fit.htm). All SEM analyses are conducted using the *lavaan* (latent variance analyses) package for R by Rosseel (2012).

### 2.2. Results and discussion

Participants obtained a general mean ITP score of *M* = 34.01, with a standard deviation of SD = 17.41. For the four ITP scenario scores on education (*M* = 43.71, SD = 17.72), healthcare (*M* = 28.50, SD = 22.62), housing (*M* = 32.65, SD = 20.40) and work (*M* = 31.16, SD = 21.32), the internal consistency reliability was *α* = 0.87 over scenarios. A CFA on a SEM model confirmed that the four ITP scenario scores on the scenarios were loading on one factor (i.e., intercultural effectiveness) as the conservative chi-squared test was not significant, *χ*^2^ (*N*_1_ = 224, 2) = 1.10, *p* = 0.58. For completeness, we also calculated the RMSEA = 0.00, 90% CI [0.000, 0.111], *p* = 0.72 (i.e., we cannot reject the hypothesis that the RMSEA value is below 0.05), the SRMR = 0.01, and the CFI = 1.00. The factor loadings read 0.78, 0.74, 0.80, and 0.85 for the education, health care, housing, and work scenario, respectively. These loadings already indicate that people tend to respond in similar fashion to the different scenarios. As an intermediary conclusion, we consider DIES to show ample reliability and construct validity.

As we were interested why the ITP variable showed such a good internal consistency reliability with only four scenarios, we further explored our data in *post hoc* analyses.^3^ We conducted these analyses based on response difference scores. A response difference score is the difference between the effective and ineffective response within one scenario and one trait origin, which is considered the base interpretation of an ITP—effect (Motowidlo et al., 2006). Model PH (see also Table 1) summarizes our findings and shows an adequate to good fit, with *χ*^2^ (*N*_1_ = 224, 162) = 217.65, *p* < 0.001, RMSEA = 0.04, 90% CI [0.02, 0.05], *p* = 0.91 (i.e., we cannot reject the hypothesis that the RMSEA value is below 0.05), the SRMR = 0.05, and the CFI = 0.97. Table 1 shows that the ITP that emerges over scenarios is the result of a focal effect of accentuating effectiveness (i.e., ITP_focal) and an additional peripheral effect of the emotional stability trait origin. Note that there were traces of other peripheral trait origin effects, but not as substantial as the emotional stability one. The endogenous *R*^2^ for response difference scores that combines both effects shows adequate results that vary from 0.30 (i.e., emotional stability in the healthcare scenario) to 0.67 (i.e., social initiative within the work scenario) with an outlier of 0.18 for cultural empathy in the work scenario. These results are in line with literature on how an ITP functions as an accentuation effect (Motowidlo et al., 2006). Indeed, the results show that a stronger focal effectiveness is linked to weaker peripheral trait origin effects and vice versa. For instance, the emotional stability difference scores do not load very high on the focal dimension, but have larger loadings on the peripheral trait origin dimension. In contrast, the other four trait origins showed high loadings on the focal dimension, but only had minor and even non-significant loadings on the peripheral trait origin dimension, to the extent the model fit deteriorated toward a non-adequate fit. As such, we have removed these peripheral trait origins from the final PH model.

**Table 1:** *Post hoc* analyses: model PH.

Latent variable	rds	*E*	SE	*z*-value	*p*	ML
**ITP_focal**						
educlat		1.00				0.89
heallat		1.07	0.15	7.08	<0.001	0.81
houslat		1.04	0.16	6.65	<0.001	0.92
worklat		0.64	0.12	5.30	<0.001	0.92
**educlat**						
	educ_ce	1.00				0.64
	educ_fx	0.83	0.10	8.33	<0.001	0.68
	educ_si	0.99	0.11	8.88	<0.001	0.74
	educ_es	−0.01	0.09	−0.10	0.918	−0.01
	educ_om	0.81	0.09	8.82	<0.001	0.73
**heallat**						
	heal_ce	1.00				0.67
	heal_fx	0.97	0.10	9.67	<0.001	0.76
	heal_si	0.95	0.10	9.74	<0.001	0.77
	heal_es	0.21	0.10	2.18	0.029	0.15
	heal_om	0.93	0.09	9.95	<0.001	0.79
**houslat**						
	hous_ce	1.00				0.57
	hous_fx	0.96	0.12	8.01	<0.001	0.74
	hous_si	1.07	0.13	8.05	<0.001	0.75
	hous_es	0.08	0.09	0.90	0.370	0.06
	hous_om	1.05	0.14	7.74	<0.001	0.70
**worklat**						
	work_ce	1.00				0.42
	work_fx	1.79	0.30	5.96	<0.001	0.75
	work_si	2.05	0.34	6.10	<0.001	0.82
	work_es	0.78	0.17	4.50	<0.001	0.40
	work_om	1.70	0.29	5.98	<0.001	0.76
**ITP_peri_es**						
	educ_es	1.00				0.63
	heal_es	1.01	0.18	5.52	<0.001	0.53
	hous_es	0.97	0.16	5.97	<0.001	0.62
	work_es	0.81	0.14	5.90	<0.001	0.56
constraints						
ITP_focal~ ~ 0*ITP_peri_es						

ITP, implicit trait policy; rds, response difference score of the effective and ineffective response within one trait origin and one scenario (e.g., educ_ce = educ_ce_effective – educ_ce_ineffective); educ , education; heal, healthcare; hous, housing; ce, cultural empathy; fx, flexibility; si, social initiative; es, emotional stability; om, open mindedness.

We also applied Model PH to the other datasets *N*_2_ and *N*_3_ of the present research’s Study 2 and 3, respectively, (see also Supplemental material S2 Appendix *Post Hoc* Analyses). A similar pattern emerged for these other datasets, featuring a strong focal dimension and a peripheral emotional stability dimension. For *N*_3_ we also observed an additional peripheral cultural empathy dimension. These *post hoc* findings provide evidence that the ITP score that emerges from a scenario is indeed based on a strong focal effectiveness dimension. A scenario score thus seems to consistently capture the essential focal part of the ITP score, generating a highly consistent score over scenarios. In addition, the multi-trait scenario setup does allow the scenario ITP score to also capture peripheral trait origin effects if the focal effectiveness dimension is less strong for one or more trait origins.

## 3. Study 2: the nomological network of intercultural effectiveness

### 3.1. Methods and materials

#### 3.1.1. Data

The ethics statement of Study 2 is analog to Study 1. Study 2 ran from November to December 2020, as the questionnaire link was spread out amongst senior economy students who could participate as a part of the lectures on intercultural effective behavior. The obtained dataset features the data of *N*_2_ = 291 students, at a completion rate of 89%, of which the vast majority (i.e., about 88%) was born between 2000 and 2002. About 70% indicated a female gender orientation, 29% indicated a male gender orientation and 1% indicated a different gender orientation. About 4% of the students indicated a migration background, while 17% indicated a father or (grand)mother with a migration background. The sample thus represents the (future) target population of the overarching project.

#### 3.1.2. Measures

Means, standard deviations, and reliabilities for the present research are reported separately in the Results section.

##### 3.1.2.1. Intercultural effectiveness

Intercultural effectiveness is scored as an ITP with the DIES instrument, according to the scoring key explained above. For Study 2, participants were asked to rate the effectiveness of the responses. A high score indicates a higher intercultural effectiveness.

##### 3.1.2.2. Multicultural personality

Multicultural Personality is measured using the Short Form Multicultural Personality Questionnaire or MPQ-SF (van der Zee et al., 2013). Items probed participants to what extent statements like “is a good listener” applied to themselves, scored on a five-point Likert scale, anchored from totally not applicable to totally applicable. The five dimensions are questioned separately (eight items each), including MPQCE (cultural empathy), MPQFX (flexibility), MPQSI (social initiative), MPQES (emotional stability), and MPQOM (open mindedness). A high score indicates a higher trait level.

##### 3.1.2.3. Intercultural attitudes and world views

Intercultural attitudes and world views are measured using the ethnocentrism—ethnorelativism scale (ECER, Schelfhout et al., 2022). The six ECER items include statements like “Would you say it is generally bad or good for the economy that people come to live here from other countries?,” scored on a 10-point Likert scale, anchored from very bad to very good. A high score indicates high levels of ethnorelativism.

##### 3.1.2.4. Intercultural capabilities

Intercultural capabilities are measured using the Adapted Self-Report CQ Scale (CQ, Schwarzenthal et al., 2019), including 24 statements like “I can describe how parents treat their children in various cultures,” scored on a five-point Likert scale, anchored from strongly disagree to strongly agree. A high score indicates higher levels of intercultural capabilities.

##### 3.1.2.5. Nomological network

The nomological network was questioned to further validate the DIES instrument, with measures of convergent and divergent validity. Right wing authoritarianism (RWA) is a personality type that is submissive to authority and easily conforms, and can act aggressively in name of this authority (Duckitt et al., 2002). RWA is measured using a shortened RWA scale (van Hiel et al., 2004). RWA includes six statements like “judges are rightfully lenient toward drug users, repression would not work anyway,” scored on a five-point Likert scale, anchored from absolutely do not agree to absolutely agree. Modern Racism (MR) is described as avoiding contact with the outgroup, while practicing racism indirectly, through attacks on policy and action (Noon, 2018). MR is measured through a MR scale (McConahay et al., 1981), presenting seven statements like “ethnic discrimination against minorities is no longer a problem in our society,” on a five-point Likert scale, anchored from absolutely do not agree to absolutely agree. Social dominance orientation (SDO) is described as a disposition to accept and prefer the current hierarchy, even disregarding the position of one’s own group (Umphress et al., 2007). SDO is measured using 16 items like “some people are just more worthy than others” on a five point Likert scale, anchored from totally not agree to totally agree (Pratto et al., 1994). Motivation to respond without prejudice is measured through 10 items like “an unbiased attitude toward Arabic minorities is important for my self-image” on a five point Likert scale, anchored from totally disagree to totally agree, adapted from Plant and Devine (1998). Internal motivation (IMS) and external motivation (EMS) are measured separately, with each five items. The need for cognitive closure (NFCC) is measured through 15 items like “I dislike unpredictable situations” on a six point Likert scale, anchored from totally not agree to totally agree, adapted from Roets and van Hiel (2011). Finally, social desirability (MC-SD) is measured through 10 items like “I never hesitate to go out of my way to help someone in trouble,” on a five point Likert scale, anchored from totally do not agree to totally agree, adapted from the MC scale for social desirability (Crowne and Marlowe, 1960). A high score on these scales indicates a higher disposition.

#### 3.1.3. Analyses

A two-sided, two-sample *t*-test is performed on the mean scores of the 20 effective responses and 20 non-effective responses to confirm that individuals indeed can distinguish intercultural effectiveness. A Cohen’s *d* presents an indication of the effect size of this difference with 0.01 = very small, 0.20 = small, 0.50 = medium, 0.80 = large, 1.20 = very large, and 2.00 = huge (Sawilowsky, 2009). A correlation matrix including mean (*M*), standard deviation (SD), and reliability (*α*) is reported to investigate if DIES generates an ITP that correlates to related measures regarding intercultural effectiveness.

### 3.2. Results and discussion

#### 3.2.1. The implicit trait policy framework of intercultural effectiveness

A two-sided, two-sample *t*-test on the mean effectiveness scores of the 20 effective DIES responses (*M* = 71.10, SD = 5.01) and 20 non-effective DIES responses (*M* = 39.10, SD = 13.49) confirms that individuals indeed can distinguish intercultural effectiveness, *t* (24.15) = 9.94, *p* < 0.001, Cohen’s *d* = 3.14. The effect size is off the scales, and represents a huge difference between effective and non-effective responses even when considering the most conservative boundaries. Note that the effect is highly similar across scenarios. These results are in line with literature stating that an ITP on intercultural competence represents GDK on which behavioral responses are effective and which are not (Osmancevic et al., 2021; Shaw, 2021).

#### 3.2.2. Nomological network

Table 2 summarizes the nomological network for intercultural effectiveness. Considering convergent validity, we observe expected positive correlations for the ITP with personality traits (MPQCE and MPQOM), ethnorelativism (ECER), cultural intelligence (CQ) and the internal motivation to respond without prejudice (IMS). We also observe expected negative correlations for the ITP with modern racism (MR), right-wing authoritarianism (RWA) and social dominance orientation (SDO). Considering divergent validity, we do not observe a link for the ITP with external motivation to respond without prejudice and with the need for cognitive closure. The correlations between the ITP and some personality traits (i.e., MPQSI and MPQFX) are also not very large and thus not significant. Such findings are not uncommon, as the framework of intercultural effectiveness (Leung et al., 2014; Schelfhout et al., 2022) does not assume a direct link between personality traits and intercultural effectiveness. Remarkably, we do observe a negative correlation between the ITP and the emotional stability trait. Although not very strong, in combination with the observation from Study 1 that emotional stability deviates from the results for the other personality traits, we have to take these findings into consideration when discussing and integrating our results into literature (see also General Discussion further below). Note that the ITP score is quite robust against social desirability (MC-SD). A weak (i.e., not significant) correlation does hint at a trend, but such a trend is not uncommon as social desirability is related to GDK (Lievens and Motowidlo, 2016). Also note that there are strong, expected correlations between a number of variables. For instance, ethnorelativism (ECER) shows a strong, negative relation with modern racism (MR), social dominance orientation (SDO) and right wing authoritarianism (RWA) as these variables all express ethnocentric attitudes or world views toward intercultural interactions. Such patterns further endorse the psychometric quality of the obtained data. On a final note, we also observe that the personality dimensions are intercorrelated, which are also in line with literature (Corstjens et al., 2017).

**Table 2:** Study 2: nomological network of intercultural effectiveness.

	*M* (SD)	*α*	ITP	MPQCE	MPQFX	MPQSI	MPQES	MPQOM	ECER	CQ	RWA	SDO	MR	EMS	IMS	NFCC	MCSD
ITP	32.13 (16.50)	0.810		0.23^**^	−0.03	0.04	−0.15^*^	0.13^*^	0.49^**^	0.24^**^	−0.34^**^	−0.49**	−0.54^**^	−0.06	0.42^**^	−0.05	0.07
MPQCE	4.12 (0.45)	0.790			−0.18^**^	0.19^**^	−0.05	0.40^**^	0.150^*^	0.37^**^	0.03	−0.18**	−0.12	−0.08	0.12	−0.03	0.26^**^
MPQFX	2.64 (0.72)	0.870				0.16^**^	0.20^**^	0.05	0.16^**^	−0.04	−0.29^**^	−0.06	−0.10	−0.06	−0.06	−0.52^**^	−0.09
MPQSI	3.20 (0.69)	0.870					0.27^**^	0.28^**^	0.06	0.10	−0.11	0.01	−0.03	−0.13^*^	−0.03	−0.21^**^	−0.07
MPQES	2.95 (0.76)	0.870						0.19^**^	−0.07	−0.06	−0.00	0.18**	0.13^*^	−0.15^*^	−0.12	−0.38^**^	0.17^**^
MPQOM	3.52 (0.55)	0.870							0.35^**^	0.53^**^	−0.22^**^	−0.20**	−0.28^**^	−0.17^**^	0.21^**^	−0.25^**^	0.18^**^
EC-ER	7.16 (1.52)	0.850								0.40^**^	−0.53^**^	−0.64**	−0.72^**^	−0.12	0.47^**^	−0.22^**^	0.08
CQ	3.56 (0.42)	0.860									−0.17^**^	−0.38*	−0.38^**^	0.04	0.33^**^	−0.02	0.17^**^
RWA	2.49 (0.69)	0.790										0.51^**^	0.56^**^	0.15^*^	−0.31^**^	0.28^**^	0.14^*^
SDO	1.83 (0.60)	0.910											0.71^**^	0.09	−0.63^**^	0.00	−0.18^**^
MR	1.94 (0.70)	0.870												0.10	−0.50^**^	0.13^*^	−0.03
EMS	2.81 (0.78)	0.810													0.14^*^	0.31^**^	−0.08
IMS	3.45 (0.55)	0.780														0.06	0.13*
NFCC	3.78 (0.64)	0.840															−0.10
MCSD	3.16 (0.50)	0.610															

ITP, implicit trait policy; MPQ, multicultural personality questionnaire; CE, cultural empathy; FX, flexibility; SI, social initiative; ES, emotional stability; OM, open mindedness; ECER, ethnocentrism/ethnorelativism; CQ, cultural intelligence; RWA, right wing authoritarianism; SDO, social dominance orientation; MR, modern racism scale; EMS, external motivation to respond without prejudice; IMS, internal motivation to respond without prejudice; NFCC, need for cognitive closure; MCSD, (Marlowe – Crown) social desirability. The reported alpha for the ITP variable is calculated over the four scenarios.*p < 0.05, **p < 0.01.

Given the data patterns observed, we can confirm that the DIES instrument indeed generates results that correlate with related measures regarding intercultural effectiveness. In other words, the DIES instrument does measure intercultural effectiveness.

## 4. Study 3: do we intend to use intercultural effectiveness?

### 4.1. Methods and materials

#### 4.1.1. Data

Study 3 ran from March to June 2021, as the questionnaire link was spread out amongst (future) health care providers who could participate as a part of the lectures on intercultural effective behavior. The obtained dataset features the data of *N*_3_ = 478 students, at a completion rate of 79%, of which the vast majority (i.e., about 76%) was born between 1999 and 2001. About 76% indicated a female gender orientation, 24% indicated a male gender orientation and less than 1% indicated a different gender orientation. About 5% of the students indicated a migration background, while 14% indicated they had a father or (grand)mother with a migration background. The sample thus represents the (future) target population of the overarching project.

#### 4.1.2. Measures

The measures are equivalent to the measures from Study 2, with the following devations. (1) For the intercultural effectiveness ITP measured by DIES, participants were asked to estimate the chance if they would react in a similar fashion. (2) The nomological network was not questioned. (3) To generate a proxy for behavioral outcome through IOC (Schwarzenthal et al., 2020), we asked participants to indicate the intensity of contacts with individuals from other cultures than their own on a four-point Likert scale,^4^ anchored from “I have only vague contacts with a different cultural background” to “I have close family and friends with a different cultural background.”

#### 4.1.3. Analyses

A structural equation model (SEM) model including DIES measures of the ITP, the five personality traits of the MPQ, EC-ER, and CI is constructed, analogous to the ITP Framework of Intercultural Effectiveness from the Introduction. SEM analyses are conducted analogous to Study 1. The fit of the theoretical framework and the data is assessed.

### 4.2. Results and discussion

#### 4.2.1. Structural equation modeling

**Figure 2:**
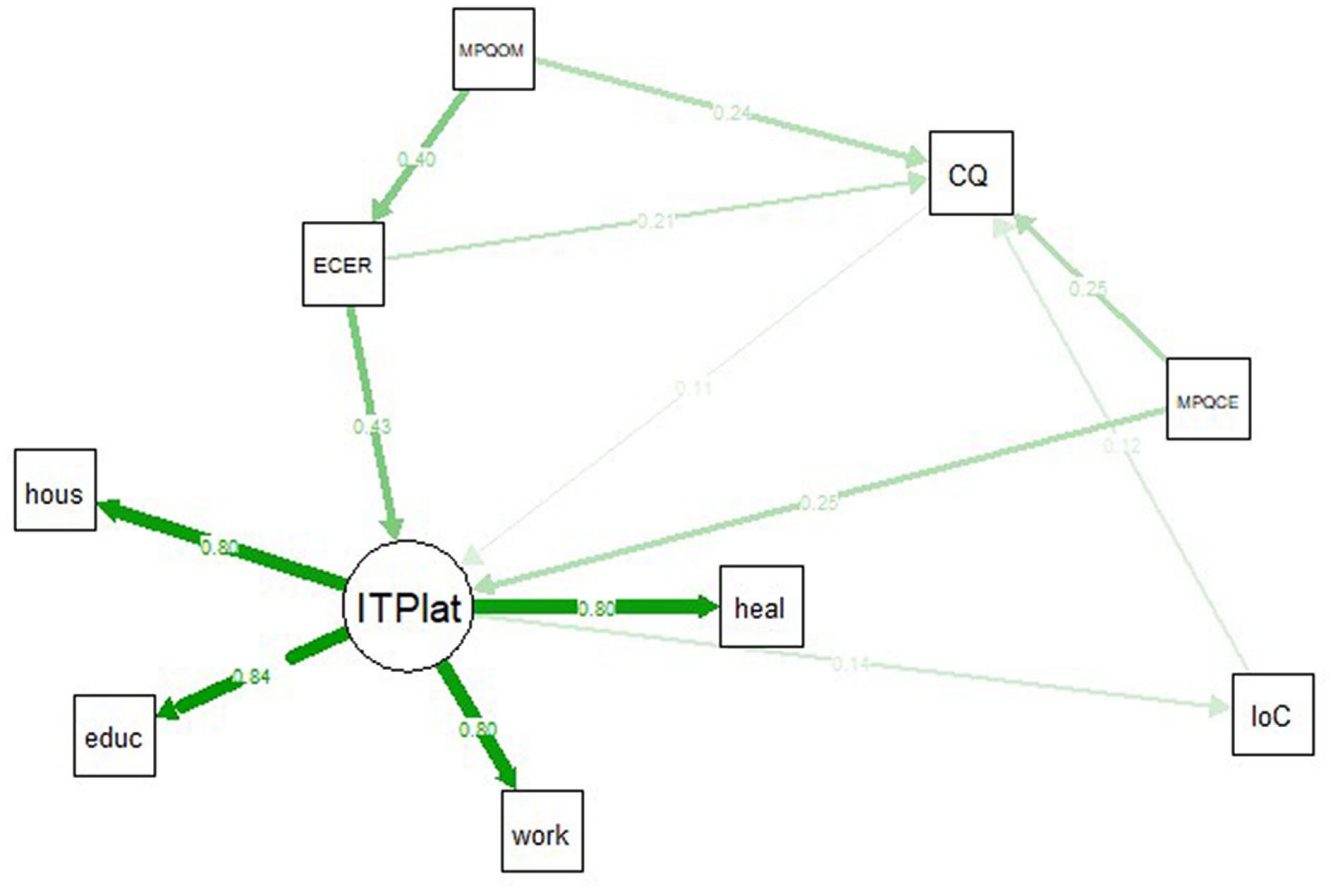
Study 3 SEM summary: the implicit trait policy framework of intercultural effectiveness. ITP, implicit trait policy; pos, positive; neg, negative; lat, latent variable; educ, score on educational scenario; heal, score on health care scenario; hous, score on housing scenario; work, score on work scenario; MPQ, multicultural personality questionnaire; CE, cultural empathy; FX, flexibility; SI, social initiative; ES, emotional stability; OM, open mindedness; ECER, ethnocentrism/ethnorelativism; CQ, cultural intelligence; IoC, intensity of contacts. The displayed figure depicts a reduced model of significant effects for reasons of clarity and parsimony. The boldness of the arrows depends on the mentioned effect sizes. ITPlat is extracted from the ITP scenario scores of education, healthcare, housing and work. ITPlat is the central node, receiving input from ECER, MPQCE, and CQ and delivering output to the IoC node. Note that the IoC node has a recursive feature and also feeds input back into CQ.

Table 3 and Figure 2 show the final SEM model, which is an operationalization of the present research’s ITP framework of intercultural effectiveness (see also Figure 1). To control for all effects present, we acted conservatively and started by including all possible effects on the ITP into the model, and not just the effects suggested by the framework of Leung et al. (2014). The final model removed the non-significant effects as these effects draw variance from the significant predictors, possibly causing an underestimation of the effects of the latter (Schelfhout et al., 2022). The final model shows an adequate fit, with *χ*^2^ (*N*_3_ = 478, 22) = 89.64, *p* < 0.001, RMSEA = 0.08, 90% CI [0.06, 0.10], *p* = 0.002, SRMR = 0.03, CFI = 0.96. The components of intercultural competence explained 35% of the variance in the latent ITP construct. Put differently, the latent ITP expressing effectiveness is saturated to a degree of *r* = 0.59 by intercultural competence. Note that the model did not require the inclusion of error variance correlations. The full analyses can be replicated as the full variance – covariance matrix is available as Supplemental material S3.

**Table 3:** Study 3 SEM summary: the implicit trait policy framework of intercultural effectiveness.

Latent	Observed	*E*	SE	*z*	*p*	ML
**ITPlat**						
	educ	1.00			<0.001	0.84
	work	1.04	0.05	19.73	<0.001	0.80
	hous	0.99	0.05	19.97	<0.001	0.80
	heal	1.03	0.05	19.83	<0.001	0.80
Dependent	Independent(s)	*E*	SE	*z*	*p*	ML
**ECER**						
	MPQOM	0.81	0.08	9.60	<0.001	0.40
**CQ**						
	MPQCE	0.60	0.10	5.86	<0.001	0.25
	MPQOM	0.52	0.10	5.33	<0.001	0.24
	EC-ER	0.22	0.05	4.93	<0.001	0.21
**ITPlat**						
	MPQCE	6.29	1.11	5.66	<0.001	0.25
	ECER	4.69	0.49	9.56	<0.001	0.43
	CQ	1.10	0.48	2.30	0.021	0.11
**IoC**						
	ITPlat	0.002	0.001	2.94	0.003	0.14
**CQ**						
	IoC	1.04	0.34	3.05	0.002	0.12

ITP, implicit trait policy; educ, DIES score on the education scenario; work, DIES score on the work scenario; hous, DIES score on the housing scenario; heal, DIES score on the healthcare scenario; MPQ, multicultural personality questionnaire; CE, cultural empathy; FX, flexibility; SI, social initiative; ES, emotional stability; OM, open mindedness; ECER, ethnocentrism/ethnorelativism; CQ, cultural intelligence; IoC, intensity of intercultural contacts. The model is trimmed by removing the non-significant effects for matters of clarity and parsimony. The full model with all effects as well as the variance—covariance matrix for replication purposes can be consulted as Supplemental material S3.

#### 4.2.2. The implicit trait policy framework of intercultural effectiveness

A two-sided, two-sample *t*-test on the mean scores of the 20 effective responses (*M* = 71.66, SD = 12.32) and 20 non-effective responses (*M* = 31.07, SD = 6.92) now confirms that individuals indeed intend to use effective behavior over ineffective behavior in intercultural situations, *t* (38) = −12.85, *p* < 0.001, Cohen’s *d* = 4.06. Again, the effect size is off the scales, and represents a huge difference between effective and non-effective responses even when considering the most conservative boundaries. Moreover, Table 3 shows that the ITP scores in all four scenarios featuring different situations show similar loadings on an underlying ITP construct, further indicating that individuals intend to use effective behavior, independent of the specific intercultural situation.

Table 3 further shows that personality predicts intercultural capabilities, both directly and indirectly. Directly, high scores on MPQCE and MPQOM lead to a higher CQ. Indirectly, high scores on the personality traits of MPQOM lead to a higher ECER score (more ethnorelative view). A higher ECER score then leads to a higher CQ score. Table 3 also shows that more intercultural capabilities (CQ) predict a higher ITP for intercultural effectiveness, while a higher ITP for intercultural effectiveness predicts a beneficial (i.e., more effective) intercultural outcome in the form of a higher intensity in intercultural contacts. For the feedback effect, Table 3 shows that a beneficial outcome in the form of a higher intensity in intercultural contacts also leads to more intercultural capabilities in the form of a higher cultural intelligence.

In sum, the judgments of the response options, the general fit indices of the SEM model, and the specific relations between the components of the SEM model all indicate that our ITP framework of intercultural effectiveness fits the empirical data, with results that are largely in line with literature on ITPs (Lievens and Motowidlo, 2016; Shaw, 2021) intercultural effectiveness (Leung et al., 2014; Schelfhout et al., 2022) and feedback (Lent et al., 1994). As such, Study 3 confirms that the DIES instrument does measure intercultural effectiveness, by directly tapping into an ITP on intercultural competence. The partial evidence regarding the trait effects needs to be addressed in the General discussion section.

## 5. General discussion

In a series of three empirical studies, the present research introduced a new instrument called the “Direct Intercultural Effectiveness Simulation” or DIES. The DIES instrument measures intercultural effectiveness, by directly tapping into an ITP on intercultural competence. The instrument was built by integrating theoretical accounts on intercultural competence (Leung et al., 2014), ITP—GDK literature (Shaw, 2021) and a feedback component from SCCT (Leung et al., 2014). Throughout these three studies, the present research revealed that our direct simulation of the ITP was reliable and valid. First, the DIES instrument reliably measures intercultural effectiveness. We consider this more than adequate reliability over scenarios a consequence of the nature of the ITP as general domain knowledge which is valid across different situations. In line with literature, the ITP that emerges from a scenario consisted of both a focal effectiveness part as well as a peripheral trait part of an ITP (Motowidlo et al., 2006). We also consider the origins of the scenarios and the responses quite representative of what is effective in specific situations, as the materials (i.e., scenarios and responses) are based on critical incidents collected from practice, further enhancing the reliability of the instrument. Second, the DIES instrument generated an ITP that showed an expected pattern when correlated with a nomological network of intercultural effectiveness, which indicated the instrument truly measures intercultural effectiveness. Third, the ITP on intercultural competence was empirically validated through integration in an ITP framework based on theoretical and empirical accounts from literature (Leung et al., 2014; Lievens and Motowidlo, 2016; Shaw, 2021; Schelfhout et al., 2022).

### 5.1. Theoretical implications for general domain knowledge and implicit trait policy literature

In general, the present research shows that the ITP construct is much more robust than previously assumed as up to 35% (or an equivalent of *r* = 0.59 as a saturation measure) of the direct ITP simulation is explained through intercultural competence. In line with the advice from literature (Ackerman, 1996; Lievens and Motowidlo, 2016; Shaw, 2021), intercultural competence as the antecedent for an ITP represents not only a full set of Big Five of human personality traits, but also intercultural attitudes and even intercultural capabilities like knowledge, motivation, and learning through cultural intelligence (Schwarzenthal et al., 2019). These numbers are higher than what is currently reported in literature regarding ITPs, with studies reporting standalone trait saturation levels rarely above *r* = 0.40 (see also Corstjens et al., 2017). On rare occasion though, more sophisticated validations use a regression of an ITP on a number of origin variables, although the reported total explained variance regarding the dependent variable is usually quite low (see Martin-Raugh et al., 2016, with an explained variance of up to 15%). The stronger validation of the ITP construct in the present research is built on the fully integrated ITP framework, directly derived from both general GDK-ITP literature (Lievens and Motowidlo, 2016) as well as specific intercultural effectiveness literature (Leung et al., 2014; Schelfhout et al., 2022). Toward future research on ITP origins, we thus advocate a similar approach in which general GDK-ITP literature is combined with work-specific literature in order to obtain a fully integrated ITP framework.

### 5.2. Theoretical implications for intercultural effectiveness literature

More specifically for intercultural effectiveness, the ITP framework integrates three (sub)processes from ITP literature (Shaw, 2021) into intercultural competence literature (Leung et al., 2014; Schelfhout et al., 2022). For the first process, individuals are able to distinguish effective from ineffective behavioral responses in intercultural situations. Individuals also indicate that they intend to use effective over ineffective behavior. Both distinction as well as intention showed huge effects. Moreover, individuals make these distinctions independent of the specific situation. These findings are in line with literature on intercultural competence (Osmancevic et al., 2021).

For the second process, personality traits indeed drive intercultural capabilities both directly and indirectly. Note that some traits did not show any effect, while emotional stability even showed negative relations with an intercultural effectiveness ITP. However, the specific implementation of a model is always function of both instruments and population (Schelfhout et al., 2022). A specific implementation over different populations can show (minor) different effects. For the present research, cultural empathy and open mindedness have the largest positive driving effect on intercultural capabilities, which is largely in line with literature (van der Zee and van Oudenhoven, 2000; van Oudenhoven and van der Zee, 2002; van der Zee et al., 2013). Regarding traits, we therefore advise caution in selecting or limiting the number of traits on which the DIES responses are based (see Limitations and future research section). First and foremost, a reaction to a situation is always affected by an individual’s full spectrum of personality, as the personality dimensions are intercorrelated (Corstjens et al., 2017). Second, the effects of personality are to a large extent indirect. Indeed, the focal difference between effective and ineffective responses is accentuated, while the peripheral trait differences within effective and ineffective items are diminished, as is also shown in literature on different topics (Tajfel, 1957; Corneille et al., 2004; Sherman et al., 2009; Dubois et al., 2010).

For the third and final process the present research showed that (intercultural) knowledge can function both as an antecedent as well as a consequence (Ackerman, 1996). In line with literature, more intercultural capabilities lead to a higher ITP, which in turn lead to more effective behavior in the form of a higher intensity of intercultural contacts (Schwarzenthal et al., 2020). Moreover, the present research explored the possibility of introducing a knowledge feedback loop into the ITP framework of intercultural effectiveness. The feedback loop was empirically verified as an increased intensity of intercultural contacts also lead to more intercultural capabilities. Combining the compelling feedback accounts in literature on SCCT (Lent et al., 1994; Stajkovic and Luthans, 1998) with the evidence from the present research, we are cautiously optimistic that future GDK and ITP research can also replicate a similar knowledge feedback loop in other settings like the work field. Besides these three processes, the ITP framework for intercultural effectiveness also showed the (expected) relation between attitudes (and world views) and capabilities (Ng et al., 2009; Young et al., 2017) and additional direct trait and attitude effects on the ITP. Indeed, open minded, empathic individuals with an ethnorelative world view displayed a higher ITP. Such more direct trait and attitude effects are already established in working literature (Lievens and Motowidlo, 2016), but we consider that these effects are as of yet not found consistently enough to warrant a change of the intercultural effectiveness model (Leung et al., 2014; Schelfhout et al., 2022).

### 5.3. Practical implications

The present research introduced the DIES instrument for practical use across intercultural contexts: a direct simulation of intercultural effectiveness by tapping into an ITP on intercultural competence. SJT—literature already contains examples of such a direct simulation (Martin-Raugh et al., 2016). Such a general instrument thus allows to make valid comparisons of intercultural effectiveness within individuals and across different situations. As such, the instrument can prove key in addressing important societal issues like prejudice and discrimination in these contexts, as these phenomena are extreme examples of (a very poor) intercultural effectiveness. Indeed, the present research clearly shows that these phenomena (see Table 2, Study 2) have moderate to even strong relations with the directly simulated ITP, which is in line with literature on intercultural effectiveness (Leung et al., 2014). Moreover, the present research also shows that the ITP is related to beneficial behavioral variables like the intensity of intercultural contacts. The ITP can thus be used to benchmark and monitor an individual’s intercultural effectiveness (possibly in combination with ITP origins and directly observable behavior) throughout awareness or training interventions addressing issues like prejudice or discrimination.

### 5.4. Strengths, limitations and future research

For sure, the DIES instrument is not the first scenario instrument that measures intercultural effectiveness (e.g., Herfst et al., 2008), nor is it the first instrument to use ITPs (Lievens and Motowidlo, 2016). To this date and to the best of our knowledge however, DIES is the first direct simulation of an ITP in the field of intercultural effectiveness, usable across different contexts like education, health care, housing, and work. Although we only explored four settings, we observed highly similar results across scenarios due to the GDK nature of the ITP. As such, we are cautiously optimistic that these results will generalize over more scenario settings. Still, future research has to confirm our assumption of generalization.

Following theoretical accounts from Shaw (2021) and Leung et al. (2014), we deviated from a rather traditional approach in measuring ITPs as for the present research ITPs were based on a full set of traits (but also attitudes and capabilities) instead of a selection of single traits only. Moreover, the scenarios functioned as a MacGuffin, while the responses were derived from traits and were presented on screen simultaneously to evoke the accentuation effect even further. Although literature does report some studies that omit the scenarios altogether (Krumm et al., 2015; Schäpers et al., 2020a), we are unaware of studies that include a full trait set to generate the answers, especially within one scenario. Still, such a setup does seem logical, as Corstjens et al. (2017) have indicated that a full set of Big Five traits is always intercorrelated (van der Zee and van Oudenhoven, 2000; van Oudenhoven and van der Zee, 2002) and can cause problems toward reliability as the specific trait-derived items generally cross-load on other traits. In other words, a reaction is never triggered by a single dimension (Corstjens et al., 2017) but can originate from all five trait dimensions (Leung et al., 2014; Schelfhout et al., 2022). We therefore consider it good practice that the instrument’s item responses are drawn up from a full set of Big Five traits for each scenario. Through the MacGuffin set up of the scenarios, participants are guided to use their own attitudes, experiences, motivation, and learning in order to judge the trait-based item responses. By combining these elements into a scenario score, the ITP score emerges as a measure of effectiveness for the specific scenario.

We consider the evidence from both the main body of research as well as the *post hoc* analyses quite compelling regarding evidence for a focal ITP dimension of effectiveness. However, we also found evidence that the emerging ITP score for a scenario can capture peripheral (trait) effects as well, if the focal accentuation for effectiveness is less present. We do acknowledge we only found limited evidence for one or possibly two peripheral trait origin effects. These trait origin effects can prove population-specific. For instance, the present research was conducted on higher education students, who showed a lower emotional stability when comparing trait scores. As the present research also features as somewhat of a pioneer study toward probing for different trait originating effects in a within scenario setup, we do call for more research on the focal vs. peripheral dichotomy using multi-trait scenarios in different populations. Future research can thus gather evidence on peripheral effects originating from specific or even all Big Five traits separately or even simultaneously.

The present research found direct effects of both trait dimensions and attitudes on the ITP variable. Although such effects are not explicitly hypothesized by original ITP models (Lievens and Motowidlo, 2016) nor by models of intercultural effectiveness (Leung et al., 2014), these models also do not exclude such effects. We therefore call for more research to clarify extra paths toward intercultural effectiveness using the ITP construct. Regarding these extra paths, future research should also consider a wider set of outcome variables for effectiveness, as our contact variable is a mere (consequential) proxy of intercultural effectiveness and not a behavioral outcome variable like ethnic discriminative behavior.

And finally, whereas the DIES was successfully validated among three different groups of future professionals (i.e., master students in sociology, economy, and health care), replication among working adults in different, work-related or training settings will further strengthen our findings.

### 5.5. Conclusion

The DIES instrument generates a reliable and valid measure of intercultural effectiveness by tapping into an ITP on intercultural competence. Theoretically, the present research integrates the instrument into literature by empirically verifying an ITP framework of intercultural effectiveness. In practice, the DIES instrument can be used as an awareness or training proxy for actual behavior to tackle important problems like ethnic prejudice and discrimination.

## Data availability statement

The datasets presented in this article are not readily available because the raw data cannot be shared due to restrictions of the ethical approval. However, the variance covariance matrix of the data is included as a Supplemental material. Requests to access the datasets should be directed to dies-tool@ugent.be.

## Ethics statement

The studies involving human participants were reviewed and approved by Institutional Review Board (or 332 Ethics Committee) of UZGent, in collaboration with Ghent University (BC-07577, 22nd of April, 2020). The patients/participants provided their online written informed consent to participate in this study.

## Author contributions

SS and ED: contributed to conception and design, contributed to acquisition of data, contributed to analysis and interpretation of data, revised the article, and approved the submitted version for publication. SS: drafted the article. All authors contributed to the article and approved the submitted version.

## Funding

The present research is part of the EdisTools project. EdisTools is funded by Research Foundation-Flanders (Strategic Basic Research – S004119N).

## Conflict of interest

The authors declare that the research was conducted in the absence of any commercial or financial relationships that could be construed as a potential conflict of interest.

## Publisher’s note

All claims expressed in this article are solely those of the authors and do not necessarily represent those of their affiliated organizations, or those of the publisher, the editors and the reviewers. Any product that may be evaluated in this article, or claim that may be made by its manufacturer, is not guaranteed or endorsed by the publisher.

## Footnotes

^1^The four scenarios were kept gender-neutral by using initials for the protagonist of the scenario.

^2^Both questions are translations from Dutch.

^3^The *post hoc* analyses were suggested by reviewer R2.

^4^Continuous and ordinal interpretations were both considered. Results were equivalent.
