# Supplementary material for: Presenting the direct intercultural effectiveness simulation: an implicit trait policy on intercultural competence
Source: Front Psychol. 2023 Jun 29;14:1137871. doi: 10.3389/fpsyg.2023.1137871 (PMC10342204; doi:10.3389/fpsyg.2023.1137871)
Supplement: Supplementary file 1 [file Data_Sheet_1.DOCX]

INFORMED CONSENT and GDPR

Before you participate in this study, we would like to point out your rights as a participant. We therefore ask you to read the text below carefully and, if applicable, to declare yourself in agreement.

I declare definitively that for a study at Ghent University:

1. I have been given an explanation of the nature of the questions that will be presented during this study and that I have been given the opportunity to obtain available information;

2. I authorize the researchers to store and process the results in a wasteful manner and report anonymously;

3. I know that I can receive a summary of the research findings upon request after the study has been completed and the results are known;

4. I consent to my data being processed by the Qualtrics software and my data being used for further analysis by other researchers after complete anonymization;

5. I know that UGent is the responsible unit with regard to personal data collected during the research and know that the data protection officer can provide me with more information about the protection of my personal information (contact xxx, masked);

6. I am aware of the processing of my personal data is based on the legal bases provided for in the General Data Protection Regulation (AVG/GDPR).

By order of the General Data Protection Regulation (or GDPR) (EU) 2016/679 of April 27, 2016, in force from May 25, 2018, on the protection of natural persons with regard to the processing of personal data and on the free movement of such data , your privacy will be observed. All information collected during this study will be processed anonymously. Confidentiality of your data is therefore always guaranteed. The data is processed and stored for a minimum of 5 years. Representatives of the client, accountants and the authorities, all bound by professional secrecy, have direct access to the procedures and data of the study, without violating the destructive power. This is only possible within the limits permitted by certain laws.
