# Supplementary material for: Presenting the direct intercultural effectiveness simulation: an implicit trait policy on intercultural competence
Source: Front Psychol. 2023 Jun 29;14:1137871. doi: 10.3389/fpsyg.2023.1137871 (PMC10342204; doi:10.3389/fpsyg.2023.1137871)
Supplement: Supplementary file 2 [file Data_Sheet_2.DOCX]

**Appendix: Posthoc Analyses**

**Premodels:** We tested different model makeups in post hoc analyses. For reasons of completeness, we provide an overview of all alternative models that have been tested before the final Model PH that is reported in main body of text. (page 1 to 30)

**Study 1, 2, & 3:** Model PH is the final post hoc model of Study 1, which we replicated in Study 2 and Study 3. (page 31 to 47)

**Premodel 0: 1 latent factor**

| Model0 =' |  |  |  |  |  |  |
| --- | --- | --- | --- | --- | --- | --- |
| + |  |  |  |  |  |  |
| + ITPlat =~ educ_ce + educ_fx + educ_si + educ_es + educ_om + |  |  |  |  |  |  |
| + heal_ce + heal_fx + heal_si + heal_es + heal_om + |  |  |  |  |  |  |
| + hous_ce + hous_fx + hous_si + hous_es + hous_om + |  |  |  |  |  |  |
| + work_ce + work_fx + work_si + work_es + work_om |  |  |  |  |  |  |
| + |  |  |  |  |  |  |
| + |  |  |  |  |  |  |
| + |  |  |  |  |  |  |
| + ' |  |  |  |  |  |  |
| > |  |  |  |  |  |  |
| > fit0 = sem(model = Model0,data = Data) |  |  |  |  |  |  |
| > summary(fit1, fit.measures = TRUE,standardized=TRUE) |  |  |  |  |  |  |
| lavaan 0.6-10 ended normally after 90 iterations |  |  |  |  |  |  |
|  |  |  |  |  |  |  |
| Estimator |  | ML |  |  |  |  |
| Optimization method |  | NLMINB |  |  |  |  |
| Number of model parameters |  | 40 |  |  |  |  |
|  |  |  |  |  |  |  |
| Number of observations |  | 224 |  |  |  |  |
|  |  |  |  |  |  |  |
| Model Test User Model: |  |  |  |  |  |  |
|  |  |  |  |  |  |  |
| Test statistic |  | 499.398 |  |  |  |  |
| Degrees of freedom |  | 170 |  |  |  |  |
| P-value (Chi-square) |  | 0 |  |  |  |  |
|  |  |  |  |  |  |  |
| Model Test Baseline Model: |  |  |  |  |  |  |
|  |  |  |  |  |  |  |
| Test statistic |  | 1946.801 |  |  |  |  |
| Degrees of freedom |  | 190 |  |  |  |  |
| P-value |  | 0 |  |  |  |  |
|  |  |  |  |  |  |  |
| User Model versus Baseline Model: |  |  |  |  |  |  |
|  |  |  |  |  |  |  |
| Comparative Fit Index (CFI) |  | 0.813 |  |  |  |  |
| Tucker-Lewis Index (TLI) |  | 0.79 |  |  |  |  |
|  |  |  |  |  |  |  |
| Loglikelihood and Information Criteria |  |  |  |  |  |  |
|  |  |  |  |  |  |  |
| Loglikelihood user model (H0) |  | -20809.1 |  |  |  |  |
| Loglikelihood unrestricted model (H1) |  | -20559.4 |  |  |  |  |
|  |  |  |  |  |  |  |
| Akaike (AIC) |  | 41698.27 |  |  |  |  |
| Bayesian (BIC) |  | 41834.74 |  |  |  |  |
| Sample-size adjusted Bayesian (BIC) |  | 41707.97 |  |  |  |  |
|  |  |  |  |  |  |  |
| Root Mean Square Error of Approximation |  |  |  |  |  |  |
|  |  |  |  |  |  |  |
| RMSEA |  | 0.093 |  |  |  |  |
| 90 Percent confidence interval - lower |  | 0.084 |  |  |  |  |
| 90 Percent confidence interval - upper |  | 0.103 |  |  |  |  |
| P-value RMSEA <= 0.05 |  | 0 |  |  |  |  |
|  |  |  |  |  |  |  |
| Standardized Root Mean Square Residual |  |  |  |  |  |  |
|  |  |  |  |  |  |  |
| SRMR |  | 0.081 |  |  |  |  |
|  |  |  |  |  |  |  |
| Parameter Estimates: |  |  |  |  |  |  |
|  |  |  |  |  |  |  |
| Standard errors |  | Standard |  |  |  |  |
| Information |  | Expected |  |  |  |  |
| Information saturated (h1) model |  | Structured |  |  |  |  |
|  |  |  |  |  |  |  |
| Latent Variables: |  |  |  |  |  |  |
|  | Estimate | Std.Err | z-value | P(>\|z\|) | Std.lv | Std.all |
| ITPlat =~ |  |  |  |  |  |  |
| educ_ce | 1 |  |  |  | 19.557 | 0.597 |
| educ_fx | 0.797 | 0.103 | 7.718 | 0 | 15.588 | 0.614 |
| educ_si | 0.907 | 0.115 | 7.906 | 0 | 17.732 | 0.634 |
| educ_es | 0.051 | 0.097 | 0.52 | 0.603 | 0.992 | 0.036 |
| educ_om | 0.792 | 0.096 | 8.224 | 0 | 15.489 | 0.669 |
| heal_ce | 1.083 | 0.147 | 7.385 | 0 | 21.177 | 0.58 |
| heal_fx | 1.02 | 0.128 | 7.973 | 0 | 19.956 | 0.641 |
| heal_si | 1.068 | 0.127 | 8.441 | 0 | 20.888 | 0.694 |
| heal_es | 0.253 | 0.12 | 2.113 | 0.035 | 4.945 | 0.148 |
| heal_om | 1.006 | 0.12 | 8.357 | 0 | 19.676 | 0.684 |
| hous_ce | 1.066 | 0.147 | 7.267 | 0 | 20.854 | 0.568 |
| hous_fx | 0.938 | 0.112 | 8.356 | 0 | 18.336 | 0.684 |
| hous_si | 1.063 | 0.125 | 8.501 | 0 | 20.781 | 0.701 |
| hous_es | 0.092 | 0.097 | 0.949 | 0.343 | 1.793 | 0.066 |
| hous_om | 1.012 | 0.128 | 7.911 | 0 | 19.788 | 0.635 |
| work_ce | 0.675 | 0.118 | 5.743 | 0 | 13.202 | 0.428 |
| work_fx | 1.075 | 0.128 | 8.376 | 0 | 21.033 | 0.686 |
| work_si | 1.257 | 0.139 | 9.032 | 0 | 24.588 | 0.765 |
| work_es | 0.499 | 0.095 | 5.226 | 0 | 9.754 | 0.385 |
| work_om | 1.036 | 0.121 | 8.533 | 0 | 20.268 | 0.704 |
|  |  |  |  |  |  |  |
| Variances: |  |  |  |  |  |  |
|  | Estimate | Std.Err | z-value | P(>\|z\|) | Std.lv | Std.all |
| .educ_ce | 689.476 | 68.216 | 10.107 | 0 | 689.476 | 0.643 |
| .educ_fx | 400.793 | 39.828 | 10.063 | 0 | 400.793 | 0.623 |
| .educ_si | 467.341 | 46.707 | 10.006 | 0 | 467.341 | 0.598 |
| .educ_es | 750.952 | 70.966 | 10.582 | 0 | 750.952 | 0.999 |
| .educ_om | 296.094 | 29.946 | 9.888 | 0 | 296.094 | 0.552 |
| .heal_ce | 883.868 | 87.098 | 10.148 | 0 | 883.868 | 0.663 |
| .heal_fx | 569.712 | 57.066 | 9.983 | 0 | 569.712 | 0.589 |
| .heal_si | 470.621 | 48.086 | 9.787 | 0 | 470.621 | 0.519 |
| .heal_es | 1089.768 | 103.161 | 10.564 | 0 | 1089.768 | 0.978 |
| .heal_om | 440.183 | 44.788 | 9.828 | 0 | 440.183 | 0.532 |
| .hous_ce | 911.117 | 89.555 | 10.174 | 0 | 911.117 | 0.677 |
| .hous_fx | 382.591 | 38.926 | 9.829 | 0 | 382.591 | 0.532 |
| .hous_si | 448.097 | 45.932 | 9.756 | 0 | 448.097 | 0.509 |
| .hous_es | 734.228 | 69.403 | 10.579 | 0 | 734.228 | 0.996 |
| .hous_om | 580.245 | 58.001 | 10.004 | 0 | 580.245 | 0.597 |
| .work_ce | 776.299 | 74.711 | 10.391 | 0 | 776.299 | 0.817 |
| .work_fx | 497.148 | 50.63 | 9.819 | 0 | 497.148 | 0.529 |
| .work_si | 428.739 | 45.751 | 9.371 | 0 | 428.739 | 0.415 |
| .work_es | 547.891 | 52.509 | 10.434 | 0 | 547.891 | 0.852 |
| .work_om | 417.274 | 42.851 | 9.738 | 0 | 417.274 | 0.504 |
| ITPlat | 382.491 | 80.153 | 4.772 | 0 | 1 | 1 |

**Premodel 1: trait model**

| > Model1 = ' |  |  |  |  |  |  |
| --- | --- | --- | --- | --- | --- | --- |
| + |  |  |  |  |  |  |
| + |  |  |  |  |  |  |
| + |  |  |  |  |  |  |
| + celat =~ educ_ce + heal_ce + hous_ce + work_ce |  |  |  |  |  |  |
| + |  |  |  |  |  |  |
| + fxlat =~ educ_fx + heal_fx + hous_fx + work_fx |  |  |  |  |  |  |
| + |  |  |  |  |  |  |
| + silat =~ educ_si + heal_si + hous_si + work_si |  |  |  |  |  |  |
| + |  |  |  |  |  |  |
| + eslat =~ educ_es + heal_es + hous_es + work_es |  |  |  |  |  |  |
| + |  |  |  |  |  |  |
| + omlat =~ educ_om + heal_om + hous_om + work_om |  |  |  |  |  |  |
| + |  |  |  |  |  |  |
| + |  |  |  |  |  |  |
| + ' |  |  |  |  |  |  |
| > |  |  |  |  |  |  |
| > fit1 = sem(model = Model1,data = Data) |  |  |  |  |  |  |
| Warning message: |  |  |  |  |  |  |
| In lav_object_post_check(object) : |  |  |  |  |  |  |
| lavaan WARNING: covariance matrix of latent variables |  |  |  |  |  |  |
| is not positive definite; |  |  |  |  |  |  |
| use lavInspect(fit, "cov.lv") to investigate. |  |  |  |  |  |  |
| > summary(fit1, fit.measures = TRUE,standardized=TRUE) |  |  |  |  |  |  |
| lavaan 0.6-10 ended normally after 335 iterations |  |  |  |  |  |  |
|  |  |  |  |  |  |  |
| Estimator |  | ML |  |  |  |  |
| Optimization method |  | NLMINB |  |  |  |  |
| Number of model parameters |  | 50 |  |  |  |  |
|  |  |  |  |  |  |  |
| Number of observations |  | 224 |  |  |  |  |
|  |  |  |  |  |  |  |
| Model Test User Model: |  |  |  |  |  |  |
|  |  |  |  |  |  |  |
| Test statistic |  | 358.317 |  |  |  |  |
| Degrees of freedom |  | 160 |  |  |  |  |
| P-value (Chi-square) |  | 0 |  |  |  |  |
|  |  |  |  |  |  |  |
| Model Test Baseline Model: |  |  |  |  |  |  |
|  |  |  |  |  |  |  |
| Test statistic |  | 1946.801 |  |  |  |  |
| Degrees of freedom |  | 190 |  |  |  |  |
| P-value |  | 0 |  |  |  |  |
|  |  |  |  |  |  |  |
| User Model versus Baseline Model: |  |  |  |  |  |  |
|  |  |  |  |  |  |  |
| Comparative Fit Index (CFI) |  | 0.887 |  |  |  |  |
| Tucker-Lewis Index (TLI) |  | 0.866 |  |  |  |  |
|  |  |  |  |  |  |  |
| Loglikelihood and Information Criteria |  |  |  |  |  |  |
|  |  |  |  |  |  |  |
| Loglikelihood user model (H0) |  | -20738.594 |  |  |  |  |
| Loglikelihood unrestricted model (H1) |  | -20559.436 |  |  |  |  |
|  |  |  |  |  |  |  |
| Akaike (AIC) |  | 41577.189 |  |  |  |  |
| Bayesian (BIC) |  | 41747.771 |  |  |  |  |
| Sample-size adjusted Bayesian (BIC) |  | 41589.313 |  |  |  |  |
|  |  |  |  |  |  |  |
| Root Mean Square Error of Approximation: |  |  |  |  |  |  |
|  |  |  |  |  |  |  |
| RMSEA |  | 0.074 |  |  |  |  |
| 90 Percent confidence interval - lower |  | 0.064 |  |  |  |  |
| 90 Percent confidence interval - upper |  | 0.085 |  |  |  |  |
| P-value RMSEA <= 0.05 |  | 0 |  |  |  |  |
|  |  |  |  |  |  |  |
| Standardized Root Mean Square Residual |  |  |  |  |  |  |
|  |  |  |  |  |  |  |
| SRMR |  | 0.073 |  |  |  |  |
|  |  |  |  |  |  |  |
| Parameter Estimates: |  |  |  |  |  |  |
|  |  |  |  |  |  |  |
| Standard errors |  | Standard |  |  |  |  |
| Information |  | Expected |  |  |  |  |
| Information saturated (h1) model |  | Structured |  |  |  |  |
|  |  |  |  |  |  |  |
| Latent Variables: |  |  |  |  |  |  |
|  | Estimate | Std.Err | z-value | P(>\|z\|) | Std.lv | Std.all |
| celat =~ |  |  |  |  |  |  |
| educ_ce | 1 |  |  |  | 20.388 | 0.623 |
| heal_ce | 1.147 | 0.15 | 7.655 | 0 | 23.376 | 0.64 |
| hous_ce | 1.116 | 0.149 | 7.472 | 0 | 22.754 | 0.62 |
| work_ce | 0.736 | 0.12 | 6.138 | 0 | 14.998 | 0.486 |
| fxlat =~ |  |  |  |  |  |  |
| educ_fx | 1 |  |  |  | 15.362 | 0.605 |
| heal_fx | 1.241 | 0.15 | 8.248 | 0 | 19.067 | 0.613 |
| hous_fx | 1.146 | 0.132 | 8.702 | 0 | 17.61 | 0.657 |
| work_fx | 1.315 | 0.151 | 8.726 | 0 | 20.209 | 0.659 |
| silat =~ |  |  |  |  |  |  |
| educ_si | 1 |  |  |  | 17.386 | 0.622 |
| heal_si | 1.175 | 0.132 | 8.893 | 0 | 20.43 | 0.678 |
| hous_si | 1.17 | 0.13 | 8.968 | 0 | 20.34 | 0.686 |
| work_si | 1.396 | 0.145 | 9.658 | 0 | 24.277 | 0.755 |
| eslat =~ |  |  |  |  |  |  |
| educ_es | 1 |  |  |  | 16.566 | 0.604 |
| heal_es | 1.096 | 0.195 | 5.633 | 0 | 18.16 | 0.544 |
| hous_es | 0.97 | 0.164 | 5.906 | 0 | 16.077 | 0.592 |
| work_es | 0.981 | 0.161 | 6.1 | 0 | 16.245 | 0.641 |
| omlat =~ |  |  |  |  |  |  |
| educ_om | 1 |  |  |  | 15.249 | 0.659 |
| heal_om | 1.266 | 0.138 | 9.191 | 0 | 19.301 | 0.671 |
| hous_om | 1.275 | 0.148 | 8.615 | 0 | 19.442 | 0.624 |
| work_om | 1.335 | 0.139 | 9.622 | 0 | 20.356 | 0.707 |
|  |  |  |  |  |  |  |
| Covariances: |  |  |  |  |  |  |
|  | Estimate | Std.Err | z-value | P(>\|z\|) | Std.lv | Std.all |
| celat ~~ |  |  |  |  |  |  |
| fxlat | 288.358 | 47.528 | 6.067 | 0 | 0.921 | 0.921 |
| silat | 328.388 | 53.372 | 6.153 | 0 | 0.926 | 0.926 |
| eslat | 73.034 | 34.313 | 2.128 | 0.033 | 0.216 | 0.216 |
| omlat | 282.194 | 45.298 | 6.23 | 0 | 0.908 | 0.908 |
| fxlat ~~ |  |  |  |  |  |  |
| silat | 295.107 | 44.506 | 6.631 | 0 | 1.105 | 1.105 |
| eslat | 59.26 | 25.43 | 2.33 | 0.02 | 0.233 | 0.233 |
| omlat | 250.303 | 37.161 | 6.736 | 0 | 1.068 | 1.068 |
| silat ~~ |  |  |  |  |  |  |
| eslat | 57.154 | 27.384 | 2.087 | 0.037 | 0.198 | 0.198 |
| omlat | 272.789 | 40.612 | 6.717 | 0 | 1.029 | 1.029 |
| eslat ~~ |  |  |  |  |  |  |
| omlat | 96.398 | 26.886 | 3.585 | 0 | 0.382 | 0.382 |
|  |  |  |  |  |  |  |
| Variances: |  |  |  |  |  |  |
|  | Estimate | Std.Err | z-value | P(>\|z\|) | Std.lv | Std.all |
| .educ_ce | 656.174 | 72.006 | 9.113 | 0 | 656.174 | 0.612 |
| .heal_ce | 785.721 | 87.786 | 8.95 | 0 | 785.721 | 0.59 |
| .hous_ce | 828.093 | 90.66 | 9.134 | 0 | 828.093 | 0.615 |
| .work_ce | 725.586 | 73.27 | 9.903 | 0 | 725.586 | 0.763 |
| .educ_fx | 408.539 | 39.272 | 10.403 | 0 | 408.539 | 0.634 |
| .heal_fx | 604.167 | 58.197 | 10.381 | 0 | 604.167 | 0.624 |
| .hous_fx | 408.476 | 39.971 | 10.219 | 0 | 408.476 | 0.568 |
| .work_fx | 530.884 | 52.007 | 10.208 | 0 | 530.884 | 0.565 |
| .educ_si | 478.08 | 46.685 | 10.241 | 0 | 478.08 | 0.613 |
| .heal_si | 489.513 | 48.71 | 10.05 | 0 | 489.513 | 0.54 |
| .hous_si | 466.188 | 46.543 | 10.016 | 0 | 466.188 | 0.53 |
| .work_si | 443.867 | 46.563 | 9.533 | 0 | 443.867 | 0.43 |
| .educ_es | 477.977 | 60.274 | 7.93 | 0 | 477.977 | 0.635 |
| .heal_es | 784.541 | 90.601 | 8.659 | 0 | 784.541 | 0.704 |
| .hous_es | 479.086 | 59.187 | 8.094 | 0 | 479.086 | 0.65 |
| .work_es | 379.24 | 51.499 | 7.364 | 0 | 379.24 | 0.59 |
| .educ_om | 303.376 | 30.523 | 9.939 | 0 | 303.376 | 0.566 |
| .heal_om | 454.716 | 46.043 | 9.876 | 0 | 454.716 | 0.55 |
| .hous_om | 593.747 | 58.876 | 10.085 | 0 | 593.747 | 0.611 |
| .work_om | 413.625 | 42.901 | 9.641 | 0 | 413.625 | 0.5 |
| celat | 415.682 | 88.057 | 4.721 | 0 | 1 | 1 |
| fxlat | 236.002 | 47.656 | 4.952 | 0 | 1 | 1 |
| silat | 302.287 | 59.458 | 5.084 | 0 | 1 | 1 |
| eslat | 274.442 | 67.883 | 4.043 | 0 | 1 | 1 |
| omlat | 232.54 | 43.041 | 5.403 | 0 | 1 | 1 |

**Premodel 2: scenarios**

| # Sceanrio model |  |  |  |  |  |  |
| --- | --- | --- | --- | --- | --- | --- |
| > Model2 = ' |  |  |  |  |  |  |
| + |  |  |  |  |  |  |
| + |  |  |  |  |  |  |
| + |  |  |  |  |  |  |
| + educlat =~ educ_ce + educ_fx + educ_si + educ_es + educ_om |  |  |  |  |  |  |
| + |  |  |  |  |  |  |
| + heallat =~ heal_ce + heal_fx + heal_si + heal_es + heal_om |  |  |  |  |  |  |
| + |  |  |  |  |  |  |
| + houslat =~ hous_ce + hous_fx + hous_si + hous_es + hous_om |  |  |  |  |  |  |
| + |  |  |  |  |  |  |
| + worklat =~ work_ce + work_fx + work_si + work_es + work_om |  |  |  |  |  |  |
| + |  |  |  |  |  |  |
| + |  |  |  |  |  |  |
| + |  |  |  |  |  |  |
| + ' |  |  |  |  |  |  |
| > |  |  |  |  |  |  |
| > fit2 = sem(model = Model2,data = Data) |  |  |  |  |  |  |
| > summary(fit2, fit.measures = TRUE,standardized=TRUE) |  |  |  |  |  |  |
| lavaan 0.6-10 ended normally after 274 iterations |  |  |  |  |  |  |
|  |  |  |  |  |  |  |
| Estimator |  | ML |  |  |  |  |
| Optimization method |  | NLMINB |  |  |  |  |
| Number of model parameters |  | 46 |  |  |  |  |
|  |  |  |  |  |  |  |
| Number of observations |  | 224 |  |  |  |  |
|  |  |  |  |  |  |  |
| Model Test User Model: |  |  |  |  |  |  |
|  |  |  |  |  |  |  |
| Test statistic |  | 346.664 |  |  |  |  |
| Degrees of freedom |  | 164 |  |  |  |  |
| P-value (Chi-square) |  | 0 |  |  |  |  |
|  |  |  |  |  |  |  |
| Model Test Baseline Model: |  |  |  |  |  |  |
|  |  |  |  |  |  |  |
| Test statistic |  | 1946.801 |  |  |  |  |
| Degrees of freedom |  | 190 |  |  |  |  |
| P-value |  | 0 |  |  |  |  |
|  |  |  |  |  |  |  |
| User Model versus Baseline Model: |  |  |  |  |  |  |
|  |  |  |  |  |  |  |
| Comparative Fit Index (CFI) |  | 0.896 |  |  |  |  |
| Tucker-Lewis Index (TLI) |  | 0.88 |  |  |  |  |
|  |  |  |  |  |  |  |
| Loglikelihood and Information Criteria |  |  |  |  |  |  |
|  |  |  |  |  |  |  |
| Loglikelihood user model (H0) |  | -20732.768 |  |  |  |  |
| Loglikelihood unrestricted model (H1) |  | -20559.436 |  |  |  |  |
|  |  |  |  |  |  |  |
| Akaike (AIC) |  | 41557.536 |  |  |  |  |
| Bayesian (BIC) |  | 41714.472 |  |  |  |  |
| Sample-size adjusted Bayesian (BIC) |  | 41568.69 |  |  |  |  |
|  |  |  |  |  |  |  |
| Root Mean Square Error of Approximation |  |  |  |  |  |  |
|  |  |  |  |  |  |  |
| RMSEA |  | 0.071 |  |  |  |  |
| 90 Percent confidence interval - lower |  | 0.06 |  |  |  |  |
| 90 Percent confidence interval - upper |  | 0.081 |  |  |  |  |
| P-value RMSEA <= 0.05 |  | 0.001 |  |  |  |  |
|  |  |  |  |  |  |  |
| Standardized Root Mean Square Residual |  |  |  |  |  |  |
|  |  |  |  |  |  |  |
| SRMR |  | 0.075 |  |  |  |  |
|  |  |  |  |  |  |  |
| Parameter Estimates: |  |  |  |  |  |  |
|  |  |  |  |  |  |  |
| Standard errors |  | Standard |  |  |  |  |
| Information |  | Expected |  |  |  |  |
| Information saturated (h1) model |  | Structured |  |  |  |  |
|  |  |  |  |  |  |  |
| Latent Variables: |  |  |  |  |  |  |
|  | Estimate | Std.Err | z-value | P(>\|z\|) | Std.lv | Std.all |
| educlat =~ |  |  |  |  |  |  |
| educ_ce | 1 |  |  |  | 20.709 | 0.632 |
| educ_fx | 0.841 | 0.101 | 8.352 | 0 | 17.425 | 0.687 |
| educ_si | 1.001 | 0.113 | 8.828 | 0 | 20.726 | 0.741 |
| educ_es | -0.016 | 0.096 | -0.163 | 0.87 | -0.323 | -0.012 |
| educ_om | 0.817 | 0.093 | 8.737 | 0 | 16.911 | 0.73 |
| heallat =~ |  |  |  |  |  |  |
| heal_ce | 1 |  |  |  | 24.507 | 0.671 |
| heal_fx | 0.964 | 0.099 | 9.697 | 0 | 23.618 | 0.759 |
| heal_si | 0.944 | 0.096 | 9.787 | 0 | 23.131 | 0.768 |
| heal_es | 0.214 | 0.098 | 2.189 | 0.029 | 5.254 | 0.157 |
| heal_om | 0.927 | 0.093 | 10.001 | 0 | 22.728 | 0.79 |
| houslat =~ |  |  |  |  |  |  |
| hous_ce | 1 |  |  |  | 21.086 | 0.575 |
| hous_fx | 0.94 | 0.116 | 8.094 | 0 | 19.812 | 0.739 |
| hous_si | 1.053 | 0.129 | 8.156 | 0 | 22.204 | 0.749 |
| hous_es | 0.09 | 0.093 | 0.976 | 0.329 | 1.906 | 0.07 |
| hous_om | 1.04 | 0.132 | 7.856 | 0 | 21.938 | 0.704 |
| worklat =~ |  |  |  |  |  |  |
| work_ce | 1 |  |  |  | 12.832 | 0.416 |
| work_fx | 1.799 | 0.303 | 5.938 | 0 | 23.09 | 0.753 |
| work_si | 2.049 | 0.337 | 6.077 | 0 | 26.289 | 0.818 |
| work_es | 0.796 | 0.179 | 4.448 | 0 | 10.211 | 0.403 |
| work_om | 1.725 | 0.289 | 5.976 | 0 | 22.14 | 0.769 |
|  |  |  |  |  |  |  |
| Covariances: |  |  |  |  |  |  |
|  | Estimate | Std.Err | z-value | P(>\|z\|) | Std.lv | Std.all |
| educlat ~~ |  |  |  |  |  |  |
| heallat | 356.859 | 62.115 | 5.745 | 0 | 0.703 | 0.703 |
| houslat | 343.563 | 61.45 | 5.591 | 0 | 0.787 | 0.787 |
| worklat | 223.637 | 46.945 | 4.764 | 0 | 0.842 | 0.842 |
| heallat ~~ |  |  |  |  |  |  |
| houslat | 409.297 | 70.904 | 5.773 | 0 | 0.792 | 0.792 |
| worklat | 225.296 | 48.173 | 4.677 | 0 | 0.716 | 0.716 |
| houslat ~~ |  |  |  |  |  |  |
| worklat | 226.756 | 49.009 | 4.627 | 0 | 0.838 | 0.838 |
|  |  |  |  |  |  |  |
| Variances: |  |  |  |  |  |  |
|  | Estimate | Std.Err | z-value | P(>\|z\|) | Std.lv | Std.all |
| .educ_ce | 643.17 | 68.609 | 9.374 | 0 | 643.17 | 0.6 |
| .educ_fx | 340.129 | 37.995 | 8.952 | 0 | 340.129 | 0.528 |
| .educ_si | 352.181 | 42.284 | 8.329 | 0 | 352.181 | 0.451 |
| .educ_es | 751.835 | 71.043 | 10.583 | 0 | 751.835 | 1 |
| .educ_om | 250.013 | 29.502 | 8.475 | 0 | 250.013 | 0.466 |
| .heal_ce | 732.299 | 79.099 | 9.258 | 0 | 732.299 | 0.549 |
| .heal_fx | 410.212 | 49.058 | 8.362 | 0 | 410.212 | 0.424 |
| .heal_si | 371.954 | 45.193 | 8.23 | 0 | 371.954 | 0.41 |
| .heal_es | 1086.594 | 103.066 | 10.543 | 0 | 1086.594 | 0.975 |
| .heal_om | 310.839 | 39.547 | 7.86 | 0 | 310.839 | 0.376 |
| .hous_ce | 900.535 | 92.163 | 9.771 | 0 | 900.535 | 0.669 |
| .hous_fx | 326.222 | 38.196 | 8.541 | 0 | 326.222 | 0.454 |
| .hous_si | 386.825 | 45.984 | 8.412 | 0 | 386.825 | 0.44 |
| .hous_es | 733.817 | 69.392 | 10.575 | 0 | 733.817 | 0.995 |
| .hous_om | 490.465 | 54.895 | 8.935 | 0 | 490.465 | 0.505 |
| .work_ce | 786.007 | 76.385 | 10.29 | 0 | 786.007 | 0.827 |
| .work_fx | 406.383 | 46.74 | 8.695 | 0 | 406.383 | 0.433 |
| .work_si | 342.168 | 44.846 | 7.63 | 0 | 342.168 | 0.331 |
| .work_es | 538.756 | 52.243 | 10.312 | 0 | 538.756 | 0.838 |
| .work_om | 337.835 | 39.807 | 8.487 | 0 | 337.835 | 0.408 |
| educlat | 428.845 | 87.07 | 4.925 | 0 | 1 | 1 |
| heallat | 600.611 | 111.998 | 5.363 | 0 | 1 | 1 |
| houslat | 444.608 | 100.836 | 4.409 | 0 | 1 | 1 |
| worklat | 164.658 | 53.589 | 3.073 | 0.002 | 1 | 1 |

**Premodel 3: traits & scenarios**

| > # Dimensions + scenarios model |  |  |  |  |  |  |
| --- | --- | --- | --- | --- | --- | --- |
| > |  |  |  |  |  |  |
| > Model3 = ' |  |  |  |  |  |  |
| + |  |  |  |  |  |  |
| + |  |  |  |  |  |  |
| + |  |  |  |  |  |  |
| + celat =~ educ_ce + heal_ce + hous_ce + work_ce |  |  |  |  |  |  |
| + |  |  |  |  |  |  |
| + fxlat =~ educ_fx + heal_fx + hous_fx + work_fx |  |  |  |  |  |  |
| + |  |  |  |  |  |  |
| + silat =~ educ_si + heal_si + hous_si + work_si |  |  |  |  |  |  |
| + |  |  |  |  |  |  |
| + eslat =~ educ_es + heal_es + hous_es + work_es |  |  |  |  |  |  |
| + |  |  |  |  |  |  |
| + omlat =~ educ_om + heal_om + hous_om + work_om |  |  |  |  |  |  |
| + |  |  |  |  |  |  |
| + |  |  |  |  |  |  |
| + |  |  |  |  |  |  |
| + educlat =~ educ_ce + educ_fx + educ_si + educ_es + educ_om |  |  |  |  |  |  |
| + |  |  |  |  |  |  |
| + heallat =~ heal_ce + heal_fx + heal_si + heal_es + heal_om |  |  |  |  |  |  |
| + |  |  |  |  |  |  |
| + houslat =~ hous_ce + hous_fx + hous_si + hous_es + hous_om |  |  |  |  |  |  |
| + |  |  |  |  |  |  |
| + worklat =~ work_ce + work_fx + work_si + work_es + work_om |  |  |  |  |  |  |
| + |  |  |  |  |  |  |
| + |  |  |  |  |  |  |
| + |  |  |  |  |  |  |
| + # constrain factor correlations between traits and situations to be zero |  |  |  |  |  |  |
| + |  |  |  |  |  |  |
| + celat~~0*educlat |  |  |  |  |  |  |
| + |  |  |  |  |  |  |
| + celat~~0*heallat |  |  |  |  |  |  |
| + |  |  |  |  |  |  |
| + celat~~0*houslat |  |  |  |  |  |  |
| + |  |  |  |  |  |  |
| + celat~~0*worklat |  |  |  |  |  |  |
| + |  |  |  |  |  |  |
| + |  |  |  |  |  |  |
| + |  |  |  |  |  |  |
| + fxlat~~0*educlat |  |  |  |  |  |  |
| + |  |  |  |  |  |  |
| + fxlat~~0*heallat |  |  |  |  |  |  |
| + |  |  |  |  |  |  |
| + fxlat~~0*houslat |  |  |  |  |  |  |
| + |  |  |  |  |  |  |
| + fxlat~~0*worklat |  |  |  |  |  |  |
| + |  |  |  |  |  |  |
| + |  |  |  |  |  |  |
| + |  |  |  |  |  |  |
| + silat~~0*educlat |  |  |  |  |  |  |
| + |  |  |  |  |  |  |
| + silat~~0*heallat |  |  |  |  |  |  |
| + |  |  |  |  |  |  |
| + silat~~0*houslat |  |  |  |  |  |  |
| + |  |  |  |  |  |  |
| + silat~~0*worklat |  |  |  |  |  |  |
| + |  |  |  |  |  |  |
| + |  |  |  |  |  |  |
| + |  |  |  |  |  |  |
| + eslat~~0*educlat |  |  |  |  |  |  |
| + |  |  |  |  |  |  |
| + eslat~~0*heallat |  |  |  |  |  |  |
| + |  |  |  |  |  |  |
| + eslat~~0*houslat |  |  |  |  |  |  |
| + |  |  |  |  |  |  |
| + eslat~~0*worklat |  |  |  |  |  |  |
| + |  |  |  |  |  |  |
| + |  |  |  |  |  |  |
| + |  |  |  |  |  |  |
| + omlat~~0*educlat |  |  |  |  |  |  |
| + |  |  |  |  |  |  |
| + omlat~~0*heallat |  |  |  |  |  |  |
| + |  |  |  |  |  |  |
| + omlat~~0*houslat |  |  |  |  |  |  |
| + |  |  |  |  |  |  |
| + omlat~~0*worklat |  |  |  |  |  |  |
| + |  |  |  |  |  |  |
| + |  |  |  |  |  |  |
| + |  |  |  |  |  |  |
| + ' |  |  |  |  |  |  |
| > fit3 = sem(model = Model3,data = Data) |  |  |  |  |  |  |
| Warning message: |  |  |  |  |  |  |
| In lav_object_post_check(object) : |  |  |  |  |  |  |
| lavaan WARNING: covariance matrix of latent variables |  |  |  |  |  |  |
| is not positive definite; |  |  |  |  |  |  |
| use lavInspect(fit, "cov.lv") to investigate. |  |  |  |  |  |  |
| > summary(fit3, fit.measures = TRUE,standardized=TRUE) |  |  |  |  |  |  |
| lavaan 0.6-10 ended normally after 808 iterations |  |  |  |  |  |  |
|  |  |  |  |  |  |  |
| Estimator |  | ML |  |  |  |  |
| Optimization method |  | NLMINB |  |  |  |  |
| Number of model parameters |  | 76 |  |  |  |  |
|  |  |  |  |  |  |  |
| Number of observations |  | 224 |  |  |  |  |
|  |  |  |  |  |  |  |
| Model Test User Model: |  |  |  |  |  |  |
|  |  |  |  |  |  |  |
| Test statistic |  | 137.526 |  |  |  |  |
| Degrees of freedom |  | 134 |  |  |  |  |
| P-value (Chi-square) |  | 0.4 |  |  |  |  |
|  |  |  |  |  |  |  |
| Model Test Baseline Model: |  |  |  |  |  |  |
|  |  |  |  |  |  |  |
| Test statistic |  | 1946.801 |  |  |  |  |
| Degrees of freedom |  | 190 |  |  |  |  |
| P-value |  | 0 |  |  |  |  |
|  |  |  |  |  |  |  |
| User Model versus Baseline Model: |  |  |  |  |  |  |
|  |  |  |  |  |  |  |
| Comparative Fit Index (CFI) |  | 0.998 |  |  |  |  |
| Tucker-Lewis Index (TLI) |  | 0.997 |  |  |  |  |
|  |  |  |  |  |  |  |
| Loglikelihood and Information Criteria |  |  |  |  |  |  |
|  |  |  |  |  |  |  |
| Loglikelihood user model (H0) |  | -20628.2 |  |  |  |  |
| Loglikelihood unrestricted model (H1) |  | -20559.4 |  |  |  |  |
|  |  |  |  |  |  |  |
| Akaike (AIC) |  | 41408.4 |  |  |  |  |
| Bayesian (BIC) |  | 41667.68 |  |  |  |  |
| Sample-size adjusted Bayesian (BIC) |  | 41426.83 |  |  |  |  |
|  |  |  |  |  |  |  |
| Root Mean Square Error of Approximation |  |  |  |  |  |  |
|  |  |  |  |  |  |  |
| RMSEA |  | 0.011 |  |  |  |  |
| 90 Percent confidence interval -lower |  | 0 |  |  |  |  |
| 90 Percent confidence interval -upper |  | 0.034 |  |  |  |  |
| P-value RMSEA <= 0.05 |  | 1 |  |  |  |  |
|  |  |  |  |  |  |  |
| Standardized Root Mean Square Residual |  |  |  |  |  |  |
|  |  |  |  |  |  |  |
| SRMR |  | 0.035 |  |  |  |  |
|  |  |  |  |  |  |  |
| Parameter Estimates: |  |  |  |  |  |  |
|  |  |  |  |  |  |  |
| Standard errors |  | Standard |  |  |  |  |
| Information |  | Expected |  |  |  |  |
| Information saturated (h1) model |  | Structured | |  |  |  |
|  |  |  |  |  |  |  |
| Latent Variables: |  |  |  |  |  |  |
|  | Estimate | Std.Err | z-value | P(>\|z\|) | Std.lv | Std.all |
| celat =~ |  |  |  |  |  |  |
| educ_ce | 1 |  |  |  | 4.932 | 0.151 |
| heal_ce | 2.965 | 1.931 | 1.535 | 0.125 | 14.623 | 0.406 |
| hous_ce | 2.634 | 1.693 | 1.556 | 0.12 | 12.992 | 0.355 |
| work_ce | 2.499 | 1.615 | 1.548 | 0.122 | 12.324 | 0.4 |
| fxlat =~ |  |  |  |  |  |  |
| educ_fx | 1 |  |  |  | 15.458 | 0.61 |
| heal_fx | 0.234 | 0.175 | 1.336 | 0.182 | 3.614 | 0.116 |
| hous_fx | 0.124 | 0.154 | 0.803 | 0.422 | 1.91 | 0.071 |
| work_fx | 0.57 | 0.225 | 2.536 | 0.011 | 8.813 | 0.287 |
| silat =~ |  |  |  |  |  |  |
| educ_si | 1 |  |  |  | 8.815 | 0.315 |
| heal_si | 0.331 | 0.241 | 1.374 | 0.169 | 2.915 | 0.097 |
| hous_si | 0.498 | 0.239 | 2.081 | 0.037 | 4.391 | 0.148 |
| work_si | 1.002 | 0.283 | 3.543 | 0 | 8.836 | 0.275 |
| eslat =~ |  |  |  |  |  |  |
| educ_es | 1 |  |  |  | 17.721 | 0.646 |
| heal_es | 1.013 | 0.177 | 5.726 | 0 | 17.959 | 0.538 |
| hous_es | 0.919 | 0.152 | 6.054 | 0 | 16.288 | 0.6 |
| work_es | 0.774 | 0.131 | 5.914 | 0 | 13.712 | 0.543 |
| omlat =~ |  |  |  |  |  |  |
| educ_om | 1 |  |  |  | 3.025 | 0.131 |
| heal_om | 1.992 | 1.363 | 1.461 | 0.144 | 6.025 | 0.209 |
| hous_om | 0.338 | 0.848 | 0.399 | 0.69 | 1.023 | 0.033 |
| work_om | 5.067 | 3.746 | 1.353 | 0.176 | 15.329 | 0.533 |
| educlat =~ |  |  |  |  |  |  |
| educ_ce | 1 |  |  |  | 21.497 | 0.656 |
| educ_fx | 0.624 | 0.112 | 5.591 | 0 | 13.422 | 0.53 |
| educ_si | 0.866 | 0.116 | 7.493 | 0 | 18.625 | 0.666 |
| educ_es | 0.01 | 0.094 | 0.112 | 0.911 | 0.226 | 0.008 |
| educ_om | 0.82 | 0.099 | 8.29 | 0 | 17.636 | 0.761 |
| heallat =~ |  |  |  |  |  |  |
| heal_ce | 1 |  |  |  | 23.216 | 0.644 |
| heal_fx | 1.008 | 0.111 | 9.055 | 0 | 23.402 | 0.752 |
| heal_si | 0.987 | 0.108 | 9.178 | 0 | 22.913 | 0.761 |
| heal_es | 0.214 | 0.102 | 2.098 | 0.036 | 4.963 | 0.149 |
| heal_om | 0.955 | 0.102 | 9.361 | 0 | 22.163 | 0.77 |
| houslat =~ |  |  |  |  |  |  |
| hous_ce | 1 |  |  |  | 20.149 | 0.55 |
| hous_fx | 0.99 | 0.131 | 7.572 | 0 | 19.954 | 0.744 |
| hous_si | 1.086 | 0.141 | 7.68 | 0 | 21.872 | 0.737 |
| hous_es | 0.11 | 0.096 | 1.145 | 0.252 | 2.225 | 0.082 |
| hous_om | 1.108 | 0.15 | 7.374 | 0 | 22.32 | 0.716 |
| worklat =~ |  |  |  |  |  |  |
| work_ce | 1 |  |  |  | 11.577 | 0.376 |
| work_fx | 1.833 | 0.372 | 4.925 | 0 | 21.215 | 0.692 |
| work_si | 2.184 | 0.423 | 5.164 | 0 | 25.289 | 0.786 |
| work_es | 0.932 | 0.231 | 4.04 | 0 | 10.793 | 0.427 |
| work_om | 1.699 | 0.34 | 5.004 | 0 | 19.671 | 0.684 |
|  |  |  |  |  |  |  |
| Covariances: |  |  |  |  |  |  |
|  | Estimate | Std.Err | z-value | P(>\|z\|) | Std.lv | Std.all |
| celat ~~ |  |  |  |  |  |  |
| educlat | 0 |  |  |  | 0 | 0 |
| heallat | 0 |  |  |  | 0 | 0 |
| houslat | 0 |  |  |  | 0 | 0 |
| worklat | 0 |  |  |  | 0 | 0 |
| fxlat ~~ |  |  |  |  |  |  |
| educlat | 0 |  |  |  | 0 | 0 |
| heallat | 0 |  |  |  | 0 | 0 |
| houslat | 0 |  |  |  | 0 | 0 |
| worklat | 0 |  |  |  | 0 | 0 |
| silat ~~ |  |  |  |  |  |  |
| educlat | 0 |  |  |  | 0 | 0 |
| heallat | 0 |  |  |  | 0 | 0 |
| houslat | 0 |  |  |  | 0 | 0 |
| worklat | 0 |  |  |  | 0 | 0 |
| eslat ~~ |  |  |  |  |  |  |
| educlat | 0 |  |  |  | 0 | 0 |
| heallat | 0 |  |  |  | 0 | 0 |
| houslat | 0 |  |  |  | 0 | 0 |
| worklat | 0 |  |  |  | 0 | 0 |
| omlat ~~ |  |  |  |  |  |  |
| educlat | 0 |  |  |  | 0 | 0 |
| heallat | 0 |  |  |  | 0 | 0 |
| houslat | 0 |  |  |  | 0 | 0 |
| worklat | 0 |  |  |  | 0 | 0 |
| celat ~~ |  |  |  |  |  |  |
| fxlat | 15.632 | 19.674 | 0.795 | 0.427 | 0.205 | 0.205 |
| silat | 13.944 | 16.724 | 0.834 | 0.404 | 0.321 | 0.321 |
| eslat | -3.072 | 11.461 | -0.268 | 0.789 | -0.035 | -0.035 |
| omlat | 3.577 | 5.442 | 0.657 | 0.511 | 0.24 | 0.24 |
| fxlat ~~ |  |  |  |  |  |  |
| silat | 154.717 | 48.983 | 3.159 | 0.002 | 1.135 | 1.135 |
| eslat | -33.483 | 34.235 | -0.978 | 0.328 | -0.122 | -0.122 |
| omlat | 33.517 | 30.625 | 1.094 | 0.274 | 0.717 | 0.717 |
| silat ~~ |  |  |  |  |  |  |
| eslat | -47.595 | 30.268 | -1.572 | 0.116 | -0.305 | -0.305 |
| omlat | 13.78 | 17.044 | 0.809 | 0.419 | 0.517 | 0.517 |
| eslat ~~ |  |  |  |  |  |  |
| omlat | 15.139 | 13.724 | 1.103 | 0.27 | 0.282 | 0.282 |
| educlat ~~ |  |  |  |  |  |  |
| heallat | 346.688 | 65.476 | 5.295 | 0 | 0.695 | 0.695 |
| houslat | 347.669 | 66.522 | 5.226 | 0 | 0.803 | 0.803 |
| worklat | 199.413 | 49.483 | 4.03 | 0 | 0.801 | 0.801 |
| heallat ~~ |  |  |  |  |  |  |
| houslat | 366.147 | 72.848 | 5.026 | 0 | 0.783 | 0.783 |
| worklat | 187.941 | 49.899 | 3.766 | 0 | 0.699 | 0.699 |
| houslat ~~ |  |  |  |  |  |  |
| worklat | 199.723 | 52.886 | 3.776 | 0 | 0.856 | 0.856 |
|  |  |  |  |  |  |  |
| Variances: |  |  |  |  |  |  |
|  | Estimate | Std.Err | z-value | P(>\|z\|) | Std.lv | Std.all |
| .educ_ce | 586.015 | 70.224 | 8.345 | 0 | 586.015 | 0.546 |
| .heal_ce | 545.692 | 106.922 | 5.104 | 0 | 545.692 | 0.42 |
| .hous_ce | 766.693 | 104.419 | 7.342 | 0 | 766.693 | 0.572 |
| .work_ce | 662.854 | 90.353 | 7.336 | 0 | 662.854 | 0.699 |
| .educ_fx | 223.104 | 81.022 | 2.754 | 0.006 | 223.104 | 0.347 |
| .heal_fx | 407.389 | 49.692 | 8.198 | 0 | 407.389 | 0.421 |
| .hous_fx | 317.71 | 38.481 | 8.256 | 0 | 317.71 | 0.442 |
| .work_fx | 412.916 | 47.825 | 8.634 | 0 | 412.916 | 0.439 |
| .educ_si | 357.873 | 45.593 | 7.849 | 0 | 357.873 | 0.457 |
| .heal_si | 371.978 | 46.342 | 8.027 | 0 | 371.978 | 0.411 |
| .hous_si | 382.793 | 45.189 | 8.471 | 0 | 382.793 | 0.435 |
| .work_si | 317.472 | 48.37 | 6.563 | 0 | 317.472 | 0.307 |
| .educ_es | 437.889 | 60.826 | 7.199 | 0 | 437.889 | 0.582 |
| .heal_es | 766.508 | 89.079 | 8.605 | 0 | 766.508 | 0.688 |
| .hous_es | 466.747 | 58.982 | 7.913 | 0 | 466.747 | 0.633 |
| .work_es | 334.132 | 44.356 | 7.533 | 0 | 334.132 | 0.523 |
| .educ_om | 216.194 | 31.425 | 6.88 | 0 | 216.194 | 0.403 |
| .heal_om | 299.949 | 39.932 | 7.511 | 0 | 299.949 | 0.363 |
| .hous_om | 473.199 | 55.85 | 8.473 | 0 | 473.199 | 0.487 |
| .work_om | 205.088 | 90.699 | 2.261 | 0.024 | 205.088 | 0.248 |
| celat | 24.326 | 29.285 | 0.831 | 0.406 | 1 | 1 |
| fxlat | 238.945 | 99.04 | 2.413 | 0.016 | 1 | 1 |
| silat | 77.703 | 51.663 | 1.504 | 0.133 | 1 | 1 |
| eslat | 314.049 | 72.973 | 4.304 | 0 | 1 | 1 |
| omlat | 9.152 | 13.3 | 0.688 | 0.491 | 1 | 1 |
| educlat | 462.138 | 94.469 | 4.892 | 0 | 1 | 1 |
| heallat | 538.971 | 108.163 | 4.983 | 0 | 1 | 1 |
| houslat | 405.964 | 99.284 | 4.089 | 0 | 1 | 1 |
| worklat | 134.029 | 52.138 | 2.571 | 0.01 | 1 | 1 |

**Premodel 4: 1 latent factor and 4 scenarios**

| > # Focal ITP + scenarios model |  |  |  |  |  |  |
| --- | --- | --- | --- | --- | --- | --- |
| > |  |  |  |  |  |  |
| > Model4 = ' |  |  |  |  |  |  |
| + |  |  |  |  |  |  |
| + |  |  |  |  |  |  |
| + |  |  |  |  |  |  |
| + ITPlat =~ educ_ce + heal_ce + hous_ce + work_ce + |  |  |  |  |  |  |
| + |  |  |  |  |  |  |
| + educ_fx + heal_fx + hous_fx + work_fx + |  |  |  |  |  |  |
| + |  |  |  |  |  |  |
| + educ_si + heal_si + hous_si + work_si + |  |  |  |  |  |  |
| + |  |  |  |  |  |  |
| + educ_es + heal_es + hous_es + work_es + |  |  |  |  |  |  |
| + |  |  |  |  |  |  |
| + educ_om + heal_om + hous_om + work_om |  |  |  |  |  |  |
| + |  |  |  |  |  |  |
| + |  |  |  |  |  |  |
| + |  |  |  |  |  |  |
| + educlat =~ educ_ce + educ_fx + educ_si + educ_es + educ_om |  |  |  |  |  |  |
| + |  |  |  |  |  |  |
| + heallat =~ heal_ce + heal_fx + heal_si + heal_es + heal_om |  |  |  |  |  |  |
| + |  |  |  |  |  |  |
| + houslat =~ hous_ce + hous_fx + hous_si + hous_es + hous_om |  |  |  |  |  |  |
| + |  |  |  |  |  |  |
| + worklat =~ work_ce + work_fx + work_si + work_es + work_om |  |  |  |  |  |  |
| + |  |  |  |  |  |  |
| + |  |  |  |  |  |  |
| + |  |  |  |  |  |  |
| + # constrain factor correlations between ITP and situations to be zero |  |  |  |  |  |  |
| + |  |  |  |  |  |  |
| + ITPlat~~0*educlat |  |  |  |  |  |  |
| + |  |  |  |  |  |  |
| + ITPlat~~0*heallat |  |  |  |  |  |  |
| + |  |  |  |  |  |  |
| + ITPlat~~0*houslat |  |  |  |  |  |  |
| + |  |  |  |  |  |  |
| + ITPlat~~0*worklat |  |  |  |  |  |  |
| + |  |  |  |  |  |  |
| + |  |  |  |  |  |  |
| + |  |  |  |  |  |  |
| + ' |  |  |  |  |  |  |
| > |  |  |  |  |  |  |
| > fit4 = sem(model = Model4,data = Data) |  |  |  |  |  |  |
| > summary(fit4, fit.measures = TRUE,standardized=TRUE) |  |  |  |  |  |  |
| lavaan 0.6-10 ended normally after 413 iterations |  |  |  |  |  |  |
|  |  |  |  |  |  |  |
| Estimator |  | ML |  |  |  |  |
| Optimization method |  | NLMINB |  |  |  |  |
| Number of model parameters |  | 66 |  |  |  |  |
|  |  |  |  |  |  |  |
| Number of observations |  | 224 |  |  |  |  |
|  |  |  |  |  |  |  |
| Model Test User Model: |  |  |  |  |  |  |
|  |  |  |  |  |  |  |
| Test statistic |  | 300.584 |  |  |  |  |
| Degrees of freedom |  | 144 |  |  |  |  |
| P-value (Chi-square) |  | 0 |  |  |  |  |
|  |  |  |  |  |  |  |
| Model Test Baseline Model: |  |  |  |  |  |  |
|  |  |  |  |  |  |  |
| Test statistic |  | 1946.801 |  |  |  |  |
| Degrees of freedom |  | 190 |  |  |  |  |
| P-value |  | 0 |  |  |  |  |
|  |  |  |  |  |  |  |
| User Model versus Baseline Model: |  |  |  |  |  |  |
|  |  |  |  |  |  |  |
| Comparative Fit Index (CFI) |  | 0.911 |  |  |  |  |
| Tucker-Lewis Index (TLI) |  | 0.882 |  |  |  |  |
|  |  |  |  |  |  |  |
| Loglikelihood and Information Criteria |  |  |  |  |  |  |
|  |  |  |  |  |  |  |
| Loglikelihood user model (H0) |  | -20709.7 |  |  |  |  |
| Loglikelihood unrestricted model (H1) |  | -20559.4 |  |  |  |  |
|  |  |  |  |  |  |  |
| Akaike (AIC) |  | 41551.46 |  |  |  |  |
| Bayesian (BIC) |  | 41776.63 |  |  |  |  |
| Sample-size adjusted Bayesian (BIC) |  | 41567.46 |  |  |  |  |
|  |  |  |  |  |  |  |
| Root Mean Square Error of Approximation |  |  |  |  |  |  |
|  |  |  |  |  |  |  |
| RMSEA |  | 0.07 |  |  |  |  |
| 90 Percent confidence interval - lower |  | 0.059 |  |  |  |  |
| 90 Percent confidence interval - upper |  | 0.081 |  |  |  |  |
| P-value RMSEA <= 0.05 |  | 0.002 |  |  |  |  |
|  |  |  |  |  |  |  |
| Standardized Root Mean Square Residual |  |  |  |  |  |  |
|  |  |  |  |  |  |  |
| SRMR |  | 0.07 |  |  |  |  |
|  |  |  |  |  |  |  |
| Parameter Estimates: |  |  |  |  |  |  |
|  |  |  |  |  |  |  |
| Standard errors |  | Standard |  |  |  |  |
| Information |  | Expected |  |  |  |  |
| Information saturated (h1) model |  | Structured | |  |  |  |
|  |  |  |  |  |  |  |
| Latent Variables: |  |  |  |  |  |  |
|  | Estimate | Std.Err | z-value | P(>\|z\|) | Std.lv | Std.all |
| ITPlat =~ |  |  |  |  |  |  |
| educ_ce | 1 |  |  |  | 13.791 | 0.421 |
| heal_ce | 2.475 | 0.543 | 4.556 | 0 | 34.137 | 0.935 |
| hous_ce | 1.428 | 0.3 | 4.751 | 0 | 19.689 | 0.537 |
| work_ce | 0.919 | 0.216 | 4.253 | 0 | 12.679 | 0.411 |
| educ_fx | 0.679 | 0.142 | 4.765 | 0 | 9.363 | 0.369 |
| heal_fx | 1.548 | 0.26 | 5.953 | 0 | 21.345 | 0.686 |
| hous_fx | 0.898 | 0.161 | 5.59 | 0 | 12.391 | 0.462 |
| work_fx | 0.872 | 0.188 | 4.639 | 0 | 12.029 | 0.392 |
| educ_si | 0.868 | 0.155 | 5.605 | 0 | 11.967 | 0.428 |
| heal_si | 1.363 | 0.24 | 5.689 | 0 | 18.793 | 0.624 |
| hous_si | 1.13 | 0.189 | 5.969 | 0 | 15.58 | 0.525 |
| work_si | 1.161 | 0.192 | 6.03 | 0 | 16.007 | 0.498 |
| educ_es | 0.029 | 0.138 | 0.212 | 0.832 | 0.403 | 0.015 |
| heal_es | 0.313 | 0.171 | 1.835 | 0.067 | 4.317 | 0.129 |
| hous_es | -0.038 | 0.147 | -0.257 | 0.797 | -0.52 | -0.019 |
| work_es | 0.521 | 0.136 | 3.822 | 0 | 7.185 | 0.283 |
| educ_om | 0.687 | 0.129 | 5.314 | 0 | 9.469 | 0.409 |
| heal_om | 1.385 | 0.22 | 6.307 | 0 | 19.1 | 0.664 |
| hous_om | 1.011 | 0.184 | 5.485 | 0 | 13.939 | 0.447 |
| work_om | 0.902 | 0.172 | 5.255 | 0 | 12.433 | 0.432 |
| educlat =~ |  |  |  |  |  |  |
| educ_ce | 1 |  |  |  | 15.123 | 0.462 |
| educ_fx | 0.989 | 0.167 | 5.932 | 0 | 14.963 | 0.59 |
| educ_si | 1.118 | 0.181 | 6.162 | 0 | 16.904 | 0.605 |
| educ_es | -0.041 | 0.138 | -0.298 | 0.766 | -0.624 | -0.023 |
| educ_om | 0.934 | 0.153 | 6.123 | 0 | 14.126 | 0.61 |
| heallat =~ |  |  |  |  |  |  |
| heal_ce | 1 |  |  |  | 8.558 | 0.234 |
| heal_fx | -1.063 | 2.255 | -0.471 | 0.637 | -9.098 | -0.292 |
| heal_si | -1.745 | 3.491 | -0.5 | 0.617 | -14.934 | -0.496 |
| heal_es | -0.327 | 0.726 | -0.45 | 0.652 | -2.797 | -0.084 |
| heal_om | -1.436 | 2.9 | -0.495 | 0.62 | -12.289 | -0.427 |
| houslat =~ |  |  |  |  |  |  |
| hous_ce | 1 |  |  |  | 9.392 | 0.256 |
| hous_fx | 1.733 | 0.545 | 3.181 | 0.001 | 16.273 | 0.607 |
| hous_si | 1.669 | 0.502 | 3.324 | 0.001 | 15.68 | 0.529 |
| hous_es | 0.38 | 0.265 | 1.435 | 0.151 | 3.572 | 0.132 |
| hous_om | 1.863 | 0.585 | 3.183 | 0.001 | 17.5 | 0.561 |
| worklat =~ |  |  |  |  |  |  |
| work_ce | 1 |  |  |  | 5.933 | 0.192 |
| work_fx | 3.405 | 1.367 | 2.49 | 0.013 | 20.2 | 0.659 |
| work_si | 3.491 | 1.361 | 2.564 | 0.01 | 20.712 | 0.644 |
| work_es | 1.23 | 0.544 | 2.26 | 0.024 | 7.3 | 0.288 |
| work_om | 3.122 | 1.24 | 2.519 | 0.012 | 18.523 | 0.644 |
|  |  |  |  |  |  |  |
| Covariances: |  |  |  |  |  |  |
|  | Estimate | Std.Err | z-value | P(>\|z\|) | Std.lv | Std.all |
| ITPlat ~~ |  |  |  |  |  |  |
| educlat | 0 |  |  |  | 0 | 0 |
| heallat | 0 |  |  |  | 0 | 0 |
| houslat | 0 |  |  |  | 0 | 0 |
| worklat | 0 |  |  |  | 0 | 0 |
| educlat ~~ |  |  |  |  |  |  |
| heallat | -67.73 | 91.216 | -0.743 | 0.458 | -0.523 | -0.523 |
| houslat | 91.388 | 52.713 | 1.734 | 0.083 | 0.643 | 0.643 |
| worklat | 67.97 | 39.92 | 1.703 | 0.089 | 0.758 | 0.758 |
| heallat ~~ |  |  |  |  |  |  |
| houslat | -41.525 | 54.024 | -0.769 | 0.442 | -0.517 | -0.517 |
| worklat | -26.548 | 34.865 | -0.761 | 0.446 | -0.523 | -0.523 |
| houslat ~~ |  |  |  |  |  |  |
| worklat | 39.923 | 29.62 | 1.348 | 0.178 | 0.716 | 0.716 |
|  |  |  |  |  |  |  |
| Variances: |  |  |  |  |  |  |
|  | Estimate | Std.Err | z-value | P(>\|z\|) | Std.lv | Std.all |
| .educ_ce | 653.143 | 68.379 | 9.552 | 0 | 653.143 | 0.609 |
| .heal_ce | 93.634 | 445.738 | 0.21 | 0.834 | 93.634 | 0.07 |
| .hous_ce | 870.184 | 90.715 | 9.593 | 0 | 870.184 | 0.646 |
| .work_ce | 754.66 | 73.67 | 10.244 | 0 | 754.66 | 0.794 |
| .educ_fx | 332.223 | 38.654 | 8.595 | 0 | 332.223 | 0.516 |
| .heal_fx | 429.509 | 49.518 | 8.674 | 0 | 429.509 | 0.444 |
| .hous_fx | 300.413 | 40.091 | 7.493 | 0 | 300.413 | 0.418 |
| .work_fx | 386.823 | 47.904 | 8.075 | 0 | 386.823 | 0.412 |
| .educ_si | 352.78 | 43.076 | 8.19 | 0 | 352.78 | 0.451 |
| .heal_si | 330.683 | 50.112 | 6.599 | 0 | 330.683 | 0.365 |
| .hous_si | 391.309 | 46.601 | 8.397 | 0 | 391.309 | 0.445 |
| .work_si | 348.076 | 45.341 | 7.677 | 0 | 348.076 | 0.337 |
| .educ_es | 751.387 | 71.014 | 10.581 | 0 | 751.387 | 0.999 |
| .heal_es | 1087.735 | 103.201 | 10.54 | 0 | 1087.735 | 0.976 |
| .hous_es | 724.42 | 68.945 | 10.507 | 0 | 724.42 | 0.982 |
| .work_es | 538.108 | 51.971 | 10.354 | 0 | 538.108 | 0.837 |
| .educ_om | 246.802 | 30.126 | 8.192 | 0 | 246.802 | 0.46 |
| .heal_om | 311.435 | 41.682 | 7.472 | 0 | 311.435 | 0.376 |
| .hous_om | 471.244 | 56.518 | 8.338 | 0 | 471.244 | 0.485 |
| .work_om | 330.351 | 40.558 | 8.145 | 0 | 330.351 | 0.399 |
| ITPlat | 190.197 | 78.572 | 2.421 | 0.015 | 1 | 1 |
| educlat | 228.695 | 84.281 | 2.713 | 0.007 | 1 | 1 |
| heallat | 73.232 | 243.838 | 0.3 | 0.764 | 1 | 1 |
| houslat | 88.217 | 62.812 | 1.404 | 0.16 | 1 | 1 |
| worklat | 35.2 | 30.706 | 1.146 | 0.252 | 1 | 1 |

**Study 1**

| > ModelITP = ' |  |  |  |  |  |  |
| --- | --- | --- | --- | --- | --- | --- |
| + |  |  |  |  |  |  |
| + |  |  |  |  |  |  |
| + ITP_focal =~ educlat + heallat + houslat + worklat |  |  |  |  |  |  |
| + |  |  |  |  |  |  |
| + educlat =~ educ_ce + educ_fx + educ_si + educ_es + educ_om |  |  |  |  |  |  |
| + |  |  |  |  |  |  |
| + heallat =~ heal_ce + heal_fx + heal_si + heal_es + heal_om |  |  |  |  |  |  |
| + |  |  |  |  |  |  |
| + houslat =~ hous_ce + hous_fx + hous_si + hous_es + hous_om |  |  |  |  |  |  |
| + |  |  |  |  |  |  |
| + worklat =~ work_ce + work_fx + work_si + work_es + work_om |  |  |  |  |  |  |
| + |  |  |  |  |  |  |
| + |  |  |  |  |  |  |
| + ITP_peri_es =~ educ_es + heal_es + hous_es + work_es |  |  |  |  |  |  |
| + |  |  |  |  |  |  |
| + # constrain factor correlations between ITP and situations to be zero |  |  |  |  |  |  |
| + |  |  |  |  |  |  |
| + ITP_focal~~0*ITP_peri_es |  |  |  |  |  |  |
| + |  |  |  |  |  |  |
| + ' |  |  |  |  |  |  |
| > |  |  |  |  |  |  |
| > fitITP = sem(model = ModelITP,data = Data) |  |  |  |  |  |  |
| > summary(fit4sex, fit.measures = TRUE,standardized=TRUE) |  |  |  |  |  |  |
| lavaan 0.6-10 ended normally after 258 iterations |  |  |  |  |  |  |
|  |  |  |  |  |  |  |
| Estimator | ML |  |  |  |  |  |
| Optimization method | NLMINB |  |  |  |  |  |
| Number of model parameters | 48 |  |  |  |  |  |
|  |  |  |  |  |  |  |
| Number of observations | 224 |  |  |  |  |  |
|  |  |  |  |  |  |  |
| Model Test User Model: |  |  |  |  |  |  |
|  |  |  |  |  |  |  |
| Test statistic | 217.648 |  |  |  |  |  |
| Degrees of freedom | 162 |  |  |  |  |  |
| P-value (Chi-square) | 0.002 |  |  |  |  |  |
|  |  |  |  |  |  |  |
| Model Test Baseline Model: |  |  |  |  |  |  |
|  |  |  |  |  |  |  |
| Test statistic | 1946.801 |  |  |  |  |  |
| Degrees of freedom | 190 |  |  |  |  |  |
| P-value | 0 |  |  |  |  |  |
|  |  |  |  |  |  |  |
| User Model versus Baseline Model: |  |  |  |  |  |  |
|  |  |  |  |  |  |  |
| Comparative Fit Index (CFI) | 0.968 |  |  |  |  |  |
| Tucker-Lewis Index (TLI) | 0.963 |  |  |  |  |  |
|  |  |  |  |  |  |  |
| Loglikelihood and Information Criteria: |  |  |  |  |  |  |
|  |  |  |  |  |  |  |
| Loglikelihood user model (H0) | -20668.26 |  |  |  |  |  |
| Loglikelihood unrestricted model (H1) | -20559.436 |  |  |  |  |  |
|  |  |  |  |  |  |  |
| Akaike (AIC) | 41432.521 |  |  |  |  |  |
| Bayesian (BIC) | 41596.28 |  |  |  |  |  |
| Sample-size adjusted Bayesian (BIC) | 41444.16 |  |  |  |  |  |
|  |  |  |  |  |  |  |
| Root Mean Square Error of Approximation: |  |  |  |  |  |  |
|  |  |  |  |  |  |  |
| RMSEA | 0.039 |  |  |  |  |  |
| 90 Percent confidence interval - lower | 0.024 |  |  |  |  |  |
| 90 Percent confidence interval - upper | 0.052 |  |  |  |  |  |
| P-value RMSEA <= 0.05 | 0.914 |  |  |  |  |  |
|  |  |  |  |  |  |  |
| Standardized Root Mean Square Residual: |  |  |  |  |  |  |
|  |  |  |  |  |  |  |
| SRMR | 0.048 |  |  |  |  |  |
|  |  |  |  |  |  |  |
| Parameter Estimates: |  |  |  |  |  |  |
|  |  |  |  |  |  |  |
| Standard errors | Standard |  |  |  |  |  |
| Information | Expected |  |  |  |  |  |
| Information saturated (h1) model | Structured |  |  |  |  |  |
|  |  |  |  |  |  |  |
| Latent Variables: |  |  |  |  |  |  |
|  | Estimate | Std.Err | z-value | P(>\|z\|) | Std.lv | Std.all |
| ITP_focal =~ |  |  |  |  |  |  |
| educlat | 1 |  |  |  | 0.885 | 0.885 |
| heallat | 1.073 | 0.152 | 7.08 | 0 | 0.811 | 0.811 |
| houslat | 1.038 | 0.156 | 6.654 | 0 | 0.92 | 0.92 |
| worklat | 0.641 | 0.121 | 5.296 | 0 | 0.919 | 0.919 |
| educlat =~ |  |  |  |  |  |  |
| educ_ce | 1 |  |  |  | 20.862 | 0.637 |
| educ_fx | 0.827 | 0.099 | 8.334 | 0 | 17.248 | 0.68 |
| educ_si | 0.992 | 0.112 | 8.876 | 0 | 20.698 | 0.74 |
| educ_es | -0.009 | 0.091 | -0.104 | 0.918 | -0.197 | -0.007 |
| educ_om | 0.814 | 0.092 | 8.817 | 0 | 16.98 | 0.733 |
| heallat =~ |  |  |  |  |  |  |
| heal_ce | 1 |  |  |  | 24.418 | 0.669 |
| heal_fx | 0.969 | 0.1 | 9.665 | 0 | 23.661 | 0.761 |
| heal_si | 0.948 | 0.097 | 9.742 | 0 | 23.137 | 0.768 |
| heal_es | 0.206 | 0.095 | 2.18 | 0.029 | 5.038 | 0.151 |
| heal_om | 0.931 | 0.094 | 9.95 | 0 | 22.726 | 0.79 |
| houslat =~ |  |  |  |  |  |  |
| hous_ce | 1 |  |  |  | 20.823 | 0.568 |
| hous_fx | 0.958 | 0.12 | 8.009 | 0 | 19.952 | 0.744 |
| hous_si | 1.07 | 0.133 | 8.049 | 0 | 22.272 | 0.751 |
| hous_es | 0.081 | 0.09 | 0.896 | 0.37 | 1.685 | 0.062 |
| hous_om | 1.051 | 0.136 | 7.737 | 0 | 21.885 | 0.702 |
| worklat =~ |  |  |  |  |  |  |
| work_ce | 1 |  |  |  | 12.88 | 0.418 |
| work_fx | 1.793 | 0.301 | 5.96 | 0 | 23.094 | 0.753 |
| work_si | 2.046 | 0.335 | 6.104 | 0 | 26.357 | 0.82 |
| work_es | 0.778 | 0.173 | 4.502 | 0 | 10.027 | 0.397 |
| work_om | 1.704 | 0.285 | 5.982 | 0 | 21.946 | 0.763 |
| ITP_peri_es =~ |  |  |  |  |  |  |
| educ_es | 1 |  |  |  | 17.389 | 0.634 |
| heal_es | 1.008 | 0.182 | 5.523 | 0 | 17.524 | 0.525 |
| hous_es | 0.97 | 0.162 | 5.968 | 0 | 16.863 | 0.621 |
| work_es | 0.81 | 0.137 | 5.895 | 0 | 14.077 | 0.557 |
|  |  |  |  |  |  |  |
| Covariances: |  |  |  |  |  |  |
|  | Estimate | Std.Err | z-value | P(>\|z\|) | Std.lv | Std.all |
| ITP_focal ~~ |  |  |  |  |  |  |
| ITP_peri_es | 0 |  |  |  | 0 | 0 |
|  |  |  |  |  |  |  |
| Variances: |  |  |  |  |  |  |
|  | Estimate | Std.Err | z-value | P(>\|z\|) | Std.lv | Std.all |
| .educ_ce | 636.748 | 68.214 | 9.334 | 0 | 636.748 | 0.594 |
| .educ_fx | 346.273 | 38.458 | 9.004 | 0 | 346.273 | 0.538 |
| .educ_si | 353.356 | 42.431 | 8.328 | 0 | 353.356 | 0.452 |
| .educ_es | 449.406 | 61.977 | 7.251 | 0 | 449.406 | 0.598 |
| .educ_om | 247.684 | 29.412 | 8.421 | 0 | 247.684 | 0.462 |
| .heal_ce | 736.076 | 79.463 | 9.263 | 0 | 736.076 | 0.552 |
| .heal_fx | 408.098 | 49.04 | 8.322 | 0 | 408.098 | 0.422 |
| .heal_si | 371.614 | 45.285 | 8.206 | 0 | 371.614 | 0.41 |
| .heal_es | 779.916 | 90.216 | 8.645 | 0 | 779.916 | 0.701 |
| .heal_om | 310.861 | 39.667 | 7.837 | 0 | 310.861 | 0.376 |
| .hous_ce | 912.393 | 93.181 | 9.792 | 0 | 912.393 | 0.678 |
| .hous_fx | 320.726 | 37.961 | 8.449 | 0 | 320.726 | 0.446 |
| .hous_si | 383.885 | 45.941 | 8.356 | 0 | 383.885 | 0.436 |
| .hous_es | 449.825 | 60.298 | 7.46 | 0 | 449.825 | 0.61 |
| .hous_om | 492.838 | 55.181 | 8.931 | 0 | 492.838 | 0.507 |
| .work_ce | 784.695 | 76.288 | 10.286 | 0 | 784.695 | 0.825 |
| .work_fx | 406.228 | 46.771 | 8.685 | 0 | 406.228 | 0.432 |
| .work_si | 338.614 | 44.719 | 7.572 | 0 | 338.614 | 0.328 |
| .work_es | 340.078 | 45.163 | 7.53 | 0 | 340.078 | 0.532 |
| .work_om | 346.43 | 40.426 | 8.57 | 0 | 346.43 | 0.418 |
| ITP_focal | 340.755 | 75.032 | 4.541 | 0 | 1 | 1 |
| .educlat | 94.467 | 30.175 | 3.131 | 0.002 | 0.217 | 0.217 |
| .heallat | 204.124 | 47.581 | 4.29 | 0 | 0.342 | 0.342 |
| .houslat | 66.262 | 27.595 | 2.401 | 0.016 | 0.153 | 0.153 |
| .worklat | 25.869 | 11.576 | 2.235 | 0.025 | 0.156 | 0.156 |
| ITP_peri_es | 302.381 | 72.665 | 4.161 | 0 | 1 | 1 |

**Study 2**

| > ModelITP = ' |  |  |  |  |  |  |
| --- | --- | --- | --- | --- | --- | --- |
| + |  |  |  |  |  |  |
| + |  |  |  |  |  |  |
| + ITP_focal =~ educlat + heallat + houslat + worklat |  |  |  |  |  |  |
| + |  |  |  |  |  |  |
| + educlat =~ educ_ce + educ_fx + educ_si + educ_es + educ_om |  |  |  |  |  |  |
| + |  |  |  |  |  |  |
| + heallat =~ heal_ce + heal_fx + heal_si + heal_es + heal_om |  |  |  |  |  |  |
| + |  |  |  |  |  |  |
| + houslat =~ hous_ce + hous_fx + hous_si + hous_es + hous_om |  |  |  |  |  |  |
| + |  |  |  |  |  |  |
| + worklat =~ work_ce + work_fx + work_si + work_es + work_om |  |  |  |  |  |  |
| + |  |  |  |  |  |  |
| + ITP_peri_es =~ educ_es + heal_es + hous_es + work_es |  |  |  |  |  |  |
| + |  |  |  |  |  |  |
| + # constrain factor correlations between ITP and situations to be zero |  |  |  |  |  |  |
| + |  |  |  |  |  |  |
| + ITP_focal~~0*ITP_peri_es |  |  |  |  |  |  |
| + ' |  |  |  |  |  |  |
| > |  |  |  |  |  |  |
| > fitITP = sem(model = ModelITP,data = Data) |  |  |  |  |  |  |
| > summary(fitITP, fit.measures = TRUE,standardized=TRUE) |  |  |  |  |  |  |
| lavaan 0.6-10 ended normally after 296 iterations |  |  |  |  |  |  |
|  |  |  |  |  |  |  |
| Estimator | ML |  |  |  |  |  |
| Optimization method | NLMINB |  |  |  |  |  |
| Number of model parameters | 48 |  |  |  |  |  |
|  |  |  |  |  |  |  |
| Number of observations | 291 |  |  |  |  |  |
|  |  |  |  |  |  |  |
| Model Test User Model: |  |  |  |  |  |  |
|  |  |  |  |  |  |  |
| Test statistic | 295.216 |  |  |  |  |  |
| Degrees of freedom | 162 |  |  |  |  |  |
| P-value (Chi-square) | 0 |  |  |  |  |  |
|  |  |  |  |  |  |  |
| Model Test Baseline Model: |  |  |  |  |  |  |
|  |  |  |  |  |  |  |
| Test statistic | 2049.447 |  |  |  |  |  |
| Degrees of freedom | 190 |  |  |  |  |  |
| P-value | 0 |  |  |  |  |  |
|  |  |  |  |  |  |  |
| User Model versus Baseline Model: |  |  |  |  |  |  |
|  |  |  |  |  |  |  |
| Comparative Fit Index (CFI) | 0.928 |  |  |  |  |  |
| Tucker-Lewis Index (TLI) | 0.916 |  |  |  |  |  |
|  |  |  |  |  |  |  |
| Loglikelihood and Information Criteria |  |  |  |  |  |  |
|  |  |  |  |  |  |  |
| Loglikelihood user model (H0) | -27254.574 |  |  |  |  |  |
| Loglikelihood unrestricted model (H1) | -27106.967 |  |  |  |  |  |
|  |  |  |  |  |  |  |
| Akaike (AIC) | 54605.149 |  |  |  |  |  |
| Bayesian (BIC) | 54781.468 |  |  |  |  |  |
| Sample-size adjusted Bayesian (BIC) | 54629.251 |  |  |  |  |  |
|  |  |  |  |  |  |  |
| Root Mean Square Error of Approximation: |  |  |  |  |  |  |
|  |  |  |  |  |  |  |
| RMSEA | 0.053 |  |  |  |  |  |
| 90 Percent confidence interval -lower | 0.043 |  |  |  |  |  |
| 90 Percent confidence interval -upper | 0.063 |  |  |  |  |  |
| P-value RMSEA <= 0.05 | 0.286 |  |  |  |  |  |
|  |  |  |  |  |  |  |
| Standardized Root Mean Square Residual: |  |  |  |  |  |  |
|  |  |  |  |  |  |  |
| SRMR | 0.056 |  |  |  |  |  |
|  |  |  |  |  |  |  |
| Parameter Estimates: |  |  |  |  |  |  |
|  |  |  |  |  |  |  |
| Standard errors | Standard |  |  |  |  |  |
| Information | Expected |  |  |  |  |  |
| Information saturated (h1) model | Structured |  |  |  |  |  |
|  |  |  |  |  |  |  |
| Latent Variables: |  |  |  |  |  |  |
|  | Estimate | Std.Err | z-value | P(>\|z\|) | Std.lv | Std.all |
| ITP_focal =~ |  |  |  |  |  |  |
| educlat | 1 |  |  |  | 0.78 | 0.78 |
| heallat | 0.979 | 0.133 | 7.362 | 0 | 0.74 | 0.74 |
| houslat | 0.583 | 0.109 | 5.349 | 0 | 0.911 | 0.911 |
| worklat | 0.336 | 0.09 | 3.711 | 0 | 0.846 | 0.846 |
| educlat =~ |  |  |  |  |  |  |
| educ_ce | 1 |  |  |  | 22.805 | 0.68 |
| educ_fx | 0.733 | 0.082 | 8.924 | 0 | 16.724 | 0.599 |
| educ_si | 1.116 | 0.1 | 11.187 | 0 | 25.442 | 0.791 |
| educ_es | 0.141 | 0.071 | 1.99 | 0.047 | 3.218 | 0.122 |
| educ_om | 0.972 | 0.088 | 10.987 | 0 | 22.171 | 0.77 |
| heallat =~ |  |  |  |  |  |  |
| heal_ce | 1 |  |  |  | 23.553 | 0.686 |
| heal_fx | 0.904 | 0.093 | 9.695 | 0 | 21.289 | 0.672 |
| heal_si | 0.835 | 0.084 | 9.991 | 0 | 19.667 | 0.697 |
| heal_es | 0.257 | 0.087 | 2.97 | 0.003 | 6.053 | 0.187 |
| heal_om | 0.997 | 0.094 | 10.581 | 0 | 23.49 | 0.754 |
| houslat =~ |  |  |  |  |  |  |
| hous_ce | 1 |  |  |  | 11.386 | 0.381 |
| hous_fx | 2.073 | 0.34 | 6.09 | 0 | 23.604 | 0.791 |
| hous_si | 2.301 | 0.381 | 6.041 | 0 | 26.194 | 0.762 |
| hous_es | -0.032 | 0.129 | -0.252 | 0.801 | -0.369 | -0.015 |
| hous_om | 1.749 | 0.301 | 5.819 | 0 | 19.919 | 0.663 |
| worklat =~ |  |  |  |  |  |  |
| work_ce | 1 |  |  |  | 7.057 | 0.252 |
| work_fx | 3.405 | 0.871 | 3.908 | 0 | 24.027 | 0.718 |
| work_si | 3.739 | 0.949 | 3.94 | 0 | 26.383 | 0.779 |
| work_es | 1.485 | 0.434 | 3.422 | 0.001 | 10.482 | 0.376 |
| work_om | 3.042 | 0.788 | 3.861 | 0 | 21.466 | 0.652 |
| ITP_peri_es =~ |  |  |  |  |  |  |
| educ_es | 1 |  |  |  | 13.603 | 0.517 |
| heal_es | 1.052 | 0.226 | 4.66 | 0 | 14.309 | 0.441 |
| hous_es | 1.162 | 0.238 | 4.891 | 0 | 15.803 | 0.656 |
| work_es | 0.828 | 0.182 | 4.55 | 0 | 11.257 | 0.403 |
|  |  |  |  |  |  |  |
| Covariances: |  |  |  |  |  |  |
|  | Estimate | Std.Err | z-value | P(>\|z\|) | Std.lv | Std.all |
| ITP_focal ~~ |  |  |  |  |  |  |
| ITP_peri_es | 0 |  |  |  | 0 | 0 |
|  |  |  |  |  |  |  |
| Variances: |  |  |  |  |  |  |
|  | Estimate | Std.Err | z-value | P(>\|z\|) | Std.lv | Std.all |
| .educ_ce | 603.218 | 59.579 | 10.125 | 0 | 603.218 | 0.537 |
| .educ_fx | 498.783 | 46.12 | 10.815 | 0 | 498.783 | 0.641 |
| .educ_si | 386.868 | 46.895 | 8.25 | 0 | 386.868 | 0.374 |
| .educ_es | 495.58 | 55.714 | 8.895 | 0 | 495.58 | 0.717 |
| .educ_om | 337.464 | 38.625 | 8.737 | 0 | 337.464 | 0.407 |
| .heal_ce | 623.805 | 63.851 | 9.77 | 0 | 623.805 | 0.529 |
| .heal_fx | 551.082 | 55.407 | 9.946 | 0 | 551.082 | 0.549 |
| .heal_si | 408.797 | 42.497 | 9.62 | 0 | 408.797 | 0.514 |
| .heal_es | 810.32 | 81.406 | 9.954 | 0 | 810.32 | 0.77 |
| .heal_om | 419.334 | 48.454 | 8.654 | 0 | 419.334 | 0.432 |
| .hous_ce | 764.843 | 65.375 | 11.699 | 0 | 764.843 | 0.855 |
| .hous_fx | 334.226 | 40.184 | 8.317 | 0 | 334.226 | 0.375 |
| .hous_si | 496.844 | 55.365 | 8.974 | 0 | 496.844 | 0.42 |
| .hous_es | 330.059 | 54.986 | 6.003 | 0 | 330.059 | 0.569 |
| .hous_om | 506.504 | 48.949 | 10.347 | 0 | 506.504 | 0.561 |
| .work_ce | 731.853 | 61.529 | 11.894 | 0 | 731.853 | 0.936 |
| .work_fx | 543.938 | 58.263 | 9.336 | 0 | 543.938 | 0.485 |
| .work_si | 450.28 | 55.803 | 8.069 | 0 | 450.28 | 0.393 |
| .work_es | 542.228 | 55.027 | 9.854 | 0 | 542.228 | 0.696 |
| .work_om | 621.612 | 61.046 | 10.183 | 0 | 621.612 | 0.574 |
| ITP_focal | 316.424 | 64.411 | 4.913 | 0 | 1 | 1 |
| .educlat | 203.647 | 42.53 | 4.788 | 0 | 0.392 | 0.392 |
| .heallat | 251.344 | 50.323 | 4.995 | 0 | 0.453 | 0.453 |
| .houslat | 22.14 | 10.381 | 2.133 | 0.033 | 0.171 | 0.171 |
| .worklat | 14.117 | 7.63 | 1.85 | 0.064 | 0.283 | 0.283 |
| ITP_peri_es | 185.035 | 53.912 | 3.432 | 0.001 | 1 | 1 |

**Study 3**

| > ModelITP = ' |  |  |  |  |  |  |
| --- | --- | --- | --- | --- | --- | --- |
| + |  |  |  |  |  |  |
| + ITP_focal =~ educlat + heallat + houslat + worklat |  |  |  |  |  |  |
| + |  |  |  |  |  |  |
| + educlat =~ educ_ce + educ_fx + educ_si + educ_es + educ_om |  |  |  |  |  |  |
| + |  |  |  |  |  |  |
| + heallat =~ heal_ce + heal_fx + heal_si + heal_es + heal_om |  |  |  |  |  |  |
| + |  |  |  |  |  |  |
| + houslat =~ hous_ce + hous_fx + hous_si + hous_es + hous_om |  |  |  |  |  |  |
| + |  |  |  |  |  |  |
| + worklat =~ work_ce + work_fx + work_si + work_es + work_om |  |  |  |  |  |  |
| + |  |  |  |  |  |  |
| + ITP_peri_ce =~ educ_ce + heal_ce + hous_ce + work_ce |  |  |  |  |  |  |
| + |  |  |  |  |  |  |
| + ITP_peri_es =~ educ_es + heal_es + hous_es + work_es |  |  |  |  |  |  |
| + |  |  |  |  |  |  |
| + # constrain factor correlations between ITP and situations to be zero |  |  |  |  |  |  |
| + |  |  |  |  |  |  |
| + ITP_focal~~0*ITP_peri_ce |  |  |  |  |  |  |
| + |  |  |  |  |  |  |
| + ITP_focal~~0*ITP_peri_es |  |  |  |  |  |  |
| + |  |  |  |  |  |  |
| + ' |  |  |  |  |  |  |
| > |  |  |  |  |  |  |
| > fitITP = sem(model = ModelITP,data = Data) |  |  |  |  |  |  |
| > summary(fitITP, fit.measures = TRUE,standardized=TRUE) |  |  |  |  |  |  |
| lavaan 0.6-10 ended normally after 339 iterations |  |  |  |  |  |  |
|  |  |  |  |  |  |  |
| Estimator | ML |  |  |  |  |  |
| Optimization method | NLMINB |  |  |  |  |  |
| Number of model parameters | 53 |  |  |  |  |  |
|  |  |  |  |  |  |  |
| Number of observations | 478 |  |  |  |  |  |
|  |  |  |  |  |  |  |
| Model Test User Model: |  |  |  |  |  |  |
|  |  |  |  |  |  |  |
| Test statistic | 340.818 |  |  |  |  |  |
| Degrees of freedom | 157 |  |  |  |  |  |
| P-value (Chi-square) | 0 |  |  |  |  |  |
|  |  |  |  |  |  |  |
| Model Test Baseline Model: |  |  |  |  |  |  |
|  |  |  |  |  |  |  |
| Test statistic | 4361.508 |  |  |  |  |  |
| Degrees of freedom | 190 |  |  |  |  |  |
| P-value | 0 |  |  |  |  |  |
|  |  |  |  |  |  |  |
| User Model versus Baseline Model: |  |  |  |  |  |  |
|  |  |  |  |  |  |  |
| Comparative Fit Index (CFI) | 0.956 |  |  |  |  |  |
| Tucker-Lewis Index (TLI) | 0.947 |  |  |  |  |  |
|  |  |  |  |  |  |  |
| Loglikelihood and Information Criteria |  |  |  |  |  |  |
|  |  |  |  |  |  |  |
| Loglikelihood user model (H0) | -44153.985 |  |  |  |  |  |
| Loglikelihood unrestricted model (H1) | -43983.576 |  |  |  |  |  |
|  |  |  |  |  |  |  |
| Akaike (AIC) | 88413.97 |  |  |  |  |  |
| Bayesian (BIC) | 88634.96 |  |  |  |  |  |
| Sample-size adjusted Bayesian (BIC) | 88466.744 |  |  |  |  |  |
|  |  |  |  |  |  |  |
| Root Mean Square Error of Approximation: |  |  |  |  |  |  |
|  |  |  |  |  |  |  |
| RMSEA | 0.049 |  |  |  |  |  |
| 90 Percent confidence interval - lower | 0.042 |  |  |  |  |  |
| 90 Percent confidence interval - upper | 0.057 |  |  |  |  |  |
| P-value RMSEA <= 0.05 | 0.535 |  |  |  |  |  |
|  |  |  |  |  |  |  |
| Standardized Root Mean Square Residual: |  |  |  |  |  |  |
|  |  |  |  |  |  |  |
| SRMR | 0.039 |  |  |  |  |  |
|  |  |  |  |  |  |  |
| Parameter Estimates: |  |  |  |  |  |  |
|  |  |  |  |  |  |  |
| Standard errors | Standard |  |  |  |  |  |
| Information | Expected |  |  |  |  |  |
| Information saturated (h1) model | Structured |  |  |  |  |  |
|  |  |  |  |  |  |  |
| Latent Variables: |  |  |  |  |  |  |
|  | Estimate | Std.Err | z-value | P(>\|z\|) | Std.lv | Std.all |
| ITP_focal =~ |  |  |  |  |  |  |
| educlat | 1 |  |  |  | 0.94 | 0.94 |
| heallat | 1.01 | 0.081 | 12.529 | 0 | 0.889 | 0.889 |
| houslat | 0.732 | 0.074 | 9.942 | 0 | 0.879 | 0.879 |
| worklat | 0.585 | 0.073 | 7.993 | 0 | 0.902 | 0.902 |
| educlat =~ |  |  |  |  |  |  |
| educ_ce | 1 |  |  |  | 22.373 | 0.642 |
| educ_fx | 0.876 | 0.065 | 13.543 | 0 | 19.6 | 0.744 |
| educ_si | 1.009 | 0.074 | 13.692 | 0 | 22.565 | 0.754 |
| educ_es | 0.255 | 0.063 | 4.031 | 0 | 5.704 | 0.192 |
| educ_om | 0.893 | 0.064 | 13.946 | 0 | 19.98 | 0.773 |
| heallat =~ |  |  |  |  |  |  |
| heal_ce | 1 |  |  |  | 23.915 | 0.731 |
| heal_fx | 0.908 | 0.06 | 15.252 | 0 | 21.703 | 0.726 |
| heal_si | 0.994 | 0.058 | 17.047 | 0 | 23.767 | 0.811 |
| heal_es | 0.405 | 0.065 | 6.229 | 0 | 9.682 | 0.296 |
| heal_om | 0.847 | 0.05 | 16.951 | 0 | 20.265 | 0.806 |
| houslat =~ |  |  |  |  |  |  |
| hous_ce | 1 |  |  |  | 17.512 | 0.541 |
| hous_fx | 1.217 | 0.109 | 11.172 | 0 | 21.306 | 0.752 |
| hous_si | 1.256 | 0.118 | 10.596 | 0 | 21.986 | 0.682 |
| hous_es | 0.555 | 0.088 | 6.301 | 0 | 9.721 | 0.327 |
| hous_om | 1.344 | 0.119 | 11.269 | 0 | 23.543 | 0.765 |
| worklat =~ |  |  |  |  |  |  |
| work_ce | 1 |  |  |  | 13.655 | 0.401 |
| work_fx | 1.474 | 0.186 | 7.922 | 0 | 20.131 | 0.642 |
| work_si | 1.871 | 0.222 | 8.432 | 0 | 25.55 | 0.785 |
| work_es | 0.948 | 0.133 | 7.132 | 0 | 12.94 | 0.491 |
| work_om | 1.946 | 0.23 | 8.455 | 0 | 26.578 | 0.793 |
| ITP_peri_ce =~ |  |  |  |  |  |  |
| educ_ce | 1 |  |  |  | 6.869 | 0.197 |
| heal_ce | 1.382 | 0.491 | 2.815 | 0.005 | 9.495 | 0.29 |
| hous_ce | 1.443 | 0.526 | 2.744 | 0.006 | 9.915 | 0.306 |
| work_ce | 2.3 | 0.845 | 2.723 | 0.006 | 15.802 | 0.464 |
| ITP_peri_es =~ |  |  |  |  |  |  |
| educ_es | 1 |  |  |  | 19.383 | 0.654 |
| heal_es | 0.688 | 0.105 | 6.532 | 0 | 13.331 | 0.408 |
| hous_es | 0.906 | 0.117 | 7.726 | 0 | 17.554 | 0.59 |
| work_es | 0.608 | 0.083 | 7.296 | 0 | 11.788 | 0.447 |
|  |  |  |  |  |  |  |
| Covariances: |  |  |  |  |  |  |
|  | Estimate | Std.Err | z-value | P(>\|z\|) | Std.lv | Std.all |
| ITP_focal ~~ |  |  |  |  |  |  |
| ITP_peri_ce | 0 |  |  |  | 0 | 0 |
| ITP_peri_es | 0 |  |  |  | 0 | 0 |
| ITP_peri_ce ~~ |  |  |  |  |  |  |
| ITP_peri_es | 1.92 | 11.739 | 0.164 | 0.87 | 0.014 | 0.014 |
|  |  |  |  |  |  |  |
| Variances: |  |  |  |  |  |  |
|  | Estimate | Std.Err | z-value | P(>\|z\|) | Std.lv | Std.all |
| .educ_ce | 666.267 | 51.558 | 12.923 | 0 | 666.267 | 0.549 |
| .educ_fx | 310.731 | 24.108 | 12.889 | 0 | 310.731 | 0.447 |
| .educ_si | 385.757 | 30.358 | 12.707 | 0 | 385.757 | 0.431 |
| .educ_es | 470.609 | 54.298 | 8.667 | 0 | 470.609 | 0.535 |
| .educ_om | 268.741 | 21.771 | 12.344 | 0 | 268.741 | 0.402 |
| .heal_ce | 408.692 | 43.393 | 9.418 | 0 | 408.692 | 0.382 |
| .heal_fx | 423.484 | 31.768 | 13.331 | 0 | 423.484 | 0.473 |
| .heal_si | 294.171 | 25.057 | 11.74 | 0 | 294.171 | 0.342 |
| .heal_es | 798.409 | 59.029 | 13.526 | 0 | 798.409 | 0.746 |
| .heal_om | 221.179 | 18.642 | 11.865 | 0 | 221.179 | 0.35 |
| .hous_ce | 643.68 | 56.294 | 11.434 | 0 | 643.68 | 0.614 |
| .hous_fx | 348.952 | 28.955 | 12.052 | 0 | 348.952 | 0.435 |
| .hous_si | 556.418 | 42.108 | 13.214 | 0 | 556.418 | 0.535 |
| .hous_es | 482.451 | 49.228 | 9.8 | 0 | 482.451 | 0.545 |
| .hous_om | 392.514 | 33.408 | 11.749 | 0 | 392.514 | 0.415 |
| .work_ce | 721.267 | 95.001 | 7.592 | 0 | 721.267 | 0.623 |
| .work_fx | 577.239 | 41.696 | 13.844 | 0 | 577.239 | 0.588 |
| .work_si | 407.353 | 34.91 | 11.669 | 0 | 407.353 | 0.384 |
| .work_es | 388.54 | 32.422 | 11.984 | 0 | 388.54 | 0.559 |
| .work_om | 415.687 | 36.368 | 11.43 | 0 | 415.687 | 0.37 |
| ITP_focal | 442.588 | 62.277 | 7.107 | 0 | 1 | 1 |
| .educlat | 57.955 | 17.294 | 3.351 | 0.001 | 0.116 | 0.116 |
| .heallat | 120.245 | 21.077 | 5.705 | 0 | 0.21 | 0.21 |
| .houslat | 69.671 | 15.456 | 4.508 | 0 | 0.227 | 0.227 |
| .worklat | 34.755 | 9.938 | 3.497 | 0 | 0.186 | 0.186 |
| ITP_peri_ce | 47.186 | 27.867 | 1.693 | 0.09 | 1 | 1 |
| ITP_peri_es | 375.701 | 64.125 | 5.859 | 0 | 1 | 1 |
